# Computational pharmacogenomic screen identifies drugs that potentiate the anti-breast cancer activity of statins

**DOI:** 10.1038/s41467-022-33144-9

**Published:** 2022-10-24

**Authors:** Jenna E. van Leeuwen, Wail Ba-Alawi, Emily Branchard, Jennifer Cruickshank, Wiebke Schormann, Joseph Longo, Jennifer Silvester, Peter L. Gross, David W. Andrews, David W. Cescon, Benjamin Haibe-Kains, Linda Z. Penn, Deena M. A. Gendoo

**Affiliations:** 1 Department of Medical Biophysics, University of Toronto, 101 College Street, Toronto, ON M5G 1L7 Canada; 2 Princess Margaret Cancer Centre, University Health Network, 101 College Street, Toronto, ON M5G 1L7 Canada; 3 Biological Sciences, Sunnybrook Research Institute, University of Toronto, Toronto, ON M4N 3M5 Canada; 4 Department of Medicine, McMaster University, 1280 Main St W, Hamilton, ON L8S 4L8 Canada; 5 Division of Medical Oncology and Hematology, Department of Medicine, University of Toronto, 27 King’s College Circle, Toronto, ON M5S 1A1 Canada; 6 Department of Computer Science, University of Toronto, 10 King’s College Road, Toronto, ON M5S 3G4 Canada; 7 Ontario Institute of Cancer Research, 661 University Avenue, Suite 510, Toronto, ON M5G 0A3 Canada; 8 Centre for Computational Biology, Institute of Cancer and Genomic Sciences, University of Birmingham, Birmingham, Birmingham, B15 2TT UK; 9 Institute of Cancer and Genomic Sciences, University of Birmingham, Birmingham, Birmingham, B15 2TT UK

**Keywords:** Breast cancer, Virtual drug screening, Pharmacogenomics, Cancer genomics, Cancer therapy

## Abstract

Statins, a family of FDA-approved cholesterol-lowering drugs that inhibit the rate-limiting enzyme of the mevalonate metabolic pathway, have demonstrated anticancer activity. Evidence shows that dipyridamole potentiates statin-induced cancer cell death by blocking a restorative feedback loop triggered by statin treatment. Leveraging this knowledge, we develop an integrative pharmacogenomics pipeline to identify compounds similar to dipyridamole at the level of drug structure, cell sensitivity and molecular perturbation. To overcome the complex polypharmacology of dipyridamole, we focus our pharmacogenomics pipeline on mevalonate pathway genes, which we name mevalonate drug-network fusion (MVA-DNF). We validate top-ranked compounds, nelfinavir and honokiol, and identify that low expression of the canonical epithelial cell marker, E-cadherin, is associated with statin-compound synergy. Analysis of remaining prioritized hits led to the validation of additional compounds, clotrimazole and vemurafenib. Thus, our computational pharmacogenomic approach identifies actionable compounds with pathway-specific activities.

## Abstract (web summary)

Statins are promising for breast cancer therapy; dipyridamole can potentiate their effects, but is contraindicated in some cases. Here, the authors develop a pharmacogenomics pipeline to predict other compounds that potentiate statins, and validate the top candidates in cell line screens and 3D cultures.

## Introduction

Triple-negative breast cancer (TNBC) is an aggressive subtype of breast cancer (BC) that has a poorer prognosis compared to other major BC subtypes^1^. This poor prognosis stems from our limited understanding of the underlying biology, the relative lack of targeted therapeutics, and the associated risk of distant metastases occurring predominantly in the first two years after diagnosis^2^. Cytotoxic anthracycline and taxane-based chemotherapy regimens remain the primary option for treating TNBC, with other classes of investigational agents in various stages of development. Therefore, novel and effective therapeutics are urgently needed to combat this difficult-to-treat cancer.

**Fig. 1:**
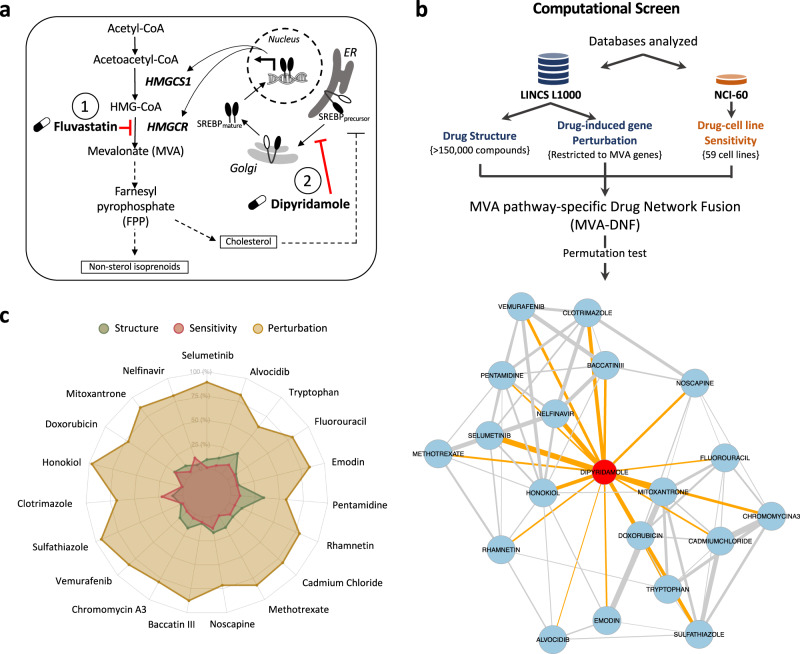
A schematic of the mevalonate (MVA) pathway and overview of the computational pharmacogenomics workflow. **a** In response to fluvastatin treatment (labeled with 1), MVA pathway end-product levels decrease, triggering an SREBP-mediated feedback response that activates MVA pathway-associated gene expression to restore cholesterol and other non-sterol end-products. Dipyridamole (DP) (labeled with 2) blocks the SREBP-mediated feedback response, thereby potentiating fluvastatin-induced cancer cell death. **b** An overview of the computational pharmacogenomics workflow, MVA-DNF, was used to identify the top 19 compounds and visualized as a compound network. MVA-DNF combines drug structure, drug-induced gene perturbation datasets restricted to six MVA pathway-specific genes, and drug sensitivity. Permutation specificity testing was performed to select compounds that have a degree of specificity to the MVA pathway and dipyridamole. Statistical significance of compounds similar to dipyridamole was assessed by comparing to 999 networks generated from random selection of six genes within the drug perturbation layer. A network representation of dipyridamole and the top 19 statistically-significant (*p*-value <0.05) compounds are shown. Each node represents a compound, and edges connect compounds based on a statistical significance cutoff of *p*-value <0.05. Blue nodes and orange edges represent the compounds connected to dipyridamole, and edge thickness represents the associated p-value between the compounds. **c** Radar plot of the top 19 compounds (*p*-value <0.05), where the contribution of each individual layer of the MVA-DNF (drug structure, perturbation, and sensitivity) is depicted. The radar plot was generated by comparing the score of DP and the top hit compound, across the affinity matrices of the perturbation, structure, and sensitivity layers. The percent contribution of each layer is shown from the center (0%) to the outer edges (100%).

Altered cellular metabolism is a hallmark of cancer^3,4^, and targeting key metabolic pathways can provide new anticancer therapeutic strategies. Aberrant activation of the mevalonate (MVA) metabolic pathway is a hallmark of many cancers, including TNBC, as the end-products include cholesterol and other non-sterol isoprenoids essential for cellular proliferation and survival^5–7^. The statin family of FDA-approved cholesterol-lowering drugs are potent inhibitors of the rate-limiting enzyme of the MVA pathway, 3-hydroxy-3-methylglutaryl-CoA reductase (HMGCR)^6^. Epidemiological evidence shows that statin use as a cholesterol control agent is associated with reduced cancer incidence^8,9^ and recurrence^10–15^. Specifically, in BC, a 30–60% reduction in recurrence is evident amongst statin users, and decreased risk is associated with increased duration of statin use^10,13,16,17^. We and others have shown preclinically that Estrogen Receptor (ER)-negative BC cell lines, including TNBC cells, are preferentially sensitive to statin-induced apoptosis^18,19^. Moreover, two preoperative clinical trials investigating lipophilic statins (fluvastatin, atorvastatin) in human BC showed statin use was associated with reduced tumor cell proliferation and increased apoptosis of high-grade BCs^20,21^. Collectively, this evidence suggests that statins have potential utility in the treatment of BC, including TNBC. While these clinical trials have been promising, there remains a gap in understanding of how best to use statins as anticancer agents. Several options include increasing the therapeutic dose of statins prescribed, or by using cholesterol-lowering doses of statins in combination with other compounds to increase the anticancer activity of statins. Drug combinations that overcome resistance mechanisms and maximize efficacy have potential advantages as cancer therapeutic strategies. This can be particularly impactful when the agents have been previously approved for use in humans, as the drug-drug combination can be fast-tracked to improve patient care. Blocking the MVA pathway with statins triggers a restorative feedback response that significantly dampens the pro-apoptotic activity of these agents^22,23^. Briefly, statin-induced depletion of intracellular sterols triggers the inactive, precursor form of the transcription factor sterol regulatory element-binding protein 2 (SREBP2) to be processed to its active, nuclear form, which induces transcription of MVA pathway genes, including *HMGCR* and the upstream synthase, 3-hydroxy-3-methylglutaryl-CoA synthase 1 (*HMGCS1*)^24^. We have shown that inhibiting SREBP2 using RNAi, or blocking SREBP2 processing using the drug dipyridamole, significantly potentiates the ability of statins to trigger tumor cell death (Fig. 1a)^23,25,26^. Dipyridamole is a FDA-approved antiplatelet agent commonly used for secondary stroke prevention, and since the statin-dipyridamole combination has been co-prescribed for other indications, it may be safely used in the treatment of cancer^27^. However, the exact mechanism of dipyridamole action remains unclear, as it has been reported to regulate several biological processes in addition to blocking SREBP-mediated feedback^28^. Moreover, the antiplatelet activity of dipyridamole may be a contraindication for some cancer patients.

Here, we show that by identifying additional compounds that potentiate the pro-apoptotic activity of statins, we bring the effective concentration of statins into a clinically achievable range and increase the utility of statins as anticancer agents in a timely manner. To this end, we developed a computational pharmacogenomics pipeline that identified compounds with similar properties to our prototypic compound, dipyridamole, at the level of structure, antiproliferative activity, and MVA pathway gene expression perturbations. By focusing our analysis on the MVA pathway, we aimed to enrich for compounds that potentiate statin-induced apoptosis. The rapid computational pharmacogenomics pipeline that we developed successfully identified several compounds that synergise with statins in both cell line and patient-derived organoid models to drive tumor cell death. Mechanistically, several of these compounds potentiated statin anticancer activity by blocking the restorative feedback response of the MVA pathway. These included nelfinavir, a FDA-approved antiretroviral drug, and honokiol, a compound originally isolated from *Magnolia spp*. We identified E-cadherin (*CDH1)* expression as a predictive biomarker of response to these combination therapies. We further evaluated the remaining hits from the in silico screen and validated additional compounds as potentiators of fluvastatin-induced cell death. Two of these blocked the feedback response, including clotrimazole, an antifungal medication, and vemurafenib, a FDA-approved BRAF V600E inhibitor. Taken together, we provide an in silico strategy to identify compounds that behave in a pathway-specific manner, and suggest this time-, cost- and labor-efficient approach will have broad utility for compound discovery across a wide variety of drug/pathway interactions.

## Results

### Computational pharmacogenomic pipeline identifies compounds that potentiate fluvastatin-induced cancer cell death and block the SREBP2-mediated feedback response

We developed a computational pharmacogenomic pipeline to identify compounds that synergise with statins by blocking the statin-induced SREBP2-mediated restorative feedback response (Fig. 1a, b). Previously, we successfully identified one agent, dipyridamole, that potentiates statins by blocking this feedback loop; however, it involved a time-, cost- and labor-intensive drug screening approach. Our goal here was to identify additional agents with these activities using an in-silico approach. Building upon our previous study^29^, we selected the LINCS-L1000 (L1000)^30^ and NCI-60^31^ datasets to mine the cellular drug response at the molecular and proliferative levels across a panel of cancer cell lines. From these datasets, we extracted drug structure, drug-cell line sensitivity profiles, and drug-induced gene perturbation data (gene expression changes after drug treatment) for 238 compounds common to both datasets. We restricted the drug-gene perturbation layer of the L1000 dataset to only include the six MVA pathway genes (*ACLY, ACAT2, HMGCS1, HMGCR, FDFT1,* and *INSIG1)* present in the L1000 landmark gene set. We took this pathway-centric approach to strategically enrich the selection of candidate compounds that possess activity targeting the MVA pathway, but not other biological processes (Supplementary Fig. 1a, b). Using dipyridamole as our reference input, we generated a MVA pathway-specific Drug Network Fusion (MVA-DNF) through the integration of three distinct data layers: drug structure, drug-cell line sensitivity profiles, and MVA gene-specific drug perturbation signatures. For each of the data layers incorporated into MVA-DNF, a 238 × 238 drug affinity matrix was generated, indicating similarity for a selected drug against all other drugs (further described in methods). Briefly, we first computed the similarity between pairs of drug structures using the Tanimoto index, prior to generating the structure affinity matrix. We computed the similarity for every pair of drug sensitivity profiles using the Pearson correlation coefficient, prior to generating an affinity matrix for the drug sensitivity layer. To create an affinity matrix for the MVA-specific perturbation layer to enrich compounds that act on the MVA pathway, we first calculated the Pearson correlation coefficient on the drug perturbation signatures that were filtered to include only MVA genes (Fig. 1b, Supplementary Fig. 1a). By integrating the three affinity matrices using similarity network fusion^32^, and filtering hits using permutation testing, we subsequently identified 19 compounds that scored as significant (permutation test *p*-value <0.05 and z-score < −1.8) (Fig. 1b, Supplementary Table 1, and further explained in methods). Represented as a network, most hits displayed strong connectivity to dipyridamole, the input compound, as well as to each other (Fig. 1b).

Since we integrated three independent layers of data (drug structure, drug-cell line sensitivity and drug-gene perturbation) to identify these compounds, we investigated the contribution of the different data layers for each of the compounds. Drug perturbation played a significant role for most compounds compared to drug sensitivity and drug structure (Fig. 1c). This reflects the specificity of the MVA-DNF towards identifying compounds that may have a role in the SREBP2-mediated feedback loop of the MVA pathway, and ultimately the potentiation of statins. Further assessment of the six MVA-pathway gene expression changes within the drug perturbation signatures highlights comparable expression perturbation profiles between dipyridamole and the identified compounds (Supplementary Fig. 1b).

To prioritize and further interrogate the 19 hits, we first excluded two compounds from further analysis as they were not clinically useful: chromomycin A3, a reported toxin^33^, and cadmium chloride, an established carcinogen^34^. The remaining 17 compounds are distributed into nine distinct categories, based on reported mechanism of action and potential clinical utility, demonstrating that the hits identified through the pharmacogenomics pipeline spanned a diverse chemical and biological space (Supplementary Fig. 1b, Supplementary Table 1). We first evaluated the top five hits for their ability to potentiate fluvastatin-induced cell death and block the SREBP2-mediated feedback response. These five compounds belonged to four different categories: RAF/MEK inhibitor (selumetinib); antiretroviral (nelfinavir); anthracycline (doxorubicin, mitoxantrone); and natural product (honokiol). The reliability of our approach is evidenced by previous work by our lab and others who have identified the lovastatin-doxorubicin combination to potentiate cancer cell death in ovarian cancer cells^35^. Moreover, RAF/MEK inhibitors such as PD98059, and more recently selumetinib (AZD6244), have been reported to synergise with statins to further potentiate cancer cell death^36,37^. Taken together, the previously reported compound (selumetinib) along with the three compounds (nelfinavir, mitoxantrone, and honokiol) were advanced for further evaluation (Supplementary Table 1).

To test whether our in silico pipeline would yield reciprocal hits, we ran the pipeline using nelfinavir and honokiol from the initial set of top drug hits. This identified dipyridamole and honokiol as top hits to nelfinavir, and identified dipyridamole and nelfinavir as top hits to honokiol (Supplementary Fig. 1c), demonstrating that the pipeline is robust and succeeds in selecting for reciprocal drug hits. To further test the robustness and stability of our algorithm, we assessed the time and memory consumption spent when running multiple iterations of MVA-DNF and permutation testing, with varying gene sizes in the drug perturbation layer (further described in methods). Our algorithm demonstrated stable processing time (CPU time in seconds) and memory consumption (in megabytes), irrespective of whether small or large numbers of genes were used to generate the pathway-centric perturbation layer (Supplementary Fig. 1d).

### Compounds identified by MVA-DNF in combination with fluvastatin induce apoptosis and block the SREBP2-regulated feedback loop of the MVA pathway

**Fig. 2:**
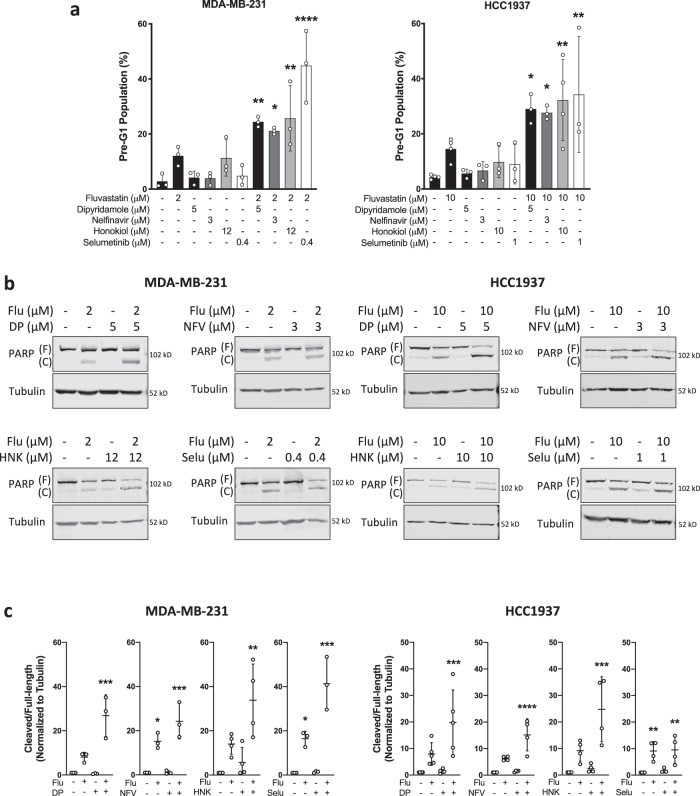
Dipyridamole-like compounds potentiate fluvastatin-induced cell death. **a** MDA-MB-231 and HCC1937 cells were treated with solvent controls or fluvastatin + /− dipyridamole (DP), nelfinavir (NFV), honokiol (HNK) or selumetinib (Selu) for 72 h, fixed in ethanol and assayed for DNA fragmentation (% pre-G1 population) as a marker of cell death by propidium iodide staining. Error bars represent the mean ± SD, *n* = 3–4 biologically independent samples, *p < 0.05, **p < 0.01, ****p < 0.0001 (one-way ANOVA with Bonferroni’s multiple comparisons test, where each treatment was compared to the solvent control). **b** Cells were treated as in (**a**), protein isolated and immunoblotting was performed to assay for PARP cleavage. Tubulin was assayed as a protein loading control. F represents full-length PARP and (C) represents cleaved PARP. **c** PARP cleavage (cleaved/full-length) shown in (**b**) was quantified by densitometry and normalized to Tubulin expression. Error bars represent the mean ± SD, *n* = 3–5 biologically independent samples, *p < 0.05, **p < 0.01, ***p < 0.001, ****p < 0.0001 (one-way ANOVA with Bonferroni’s multiple comparisons test, where each group was compared to the solvent control within each experiment). Source data are provided as source data file.

Potentiation of fluvastatin-induced cell death was evaluated by first identifying a sub-lethal compound concentration (defined here as having less than 20% cancer cell death activity alone) (Supplementary Fig. 2). After single-agent activity was assessed, sensitivity to increasing fluvastatin exposure in combination with the identified sub-lethal concentration of the compounds was evaluated in two BC cell line models (HCC1937 and MDA-MB-231). These models have differential sensitivity to fluvastatin as a single agent^18^. Fluvastatin-induced potentiation (lower IC_50_) was observed when combined with selumetinib, nelfinavir, and honokiol (Supplementary Figs. 3 and 4). Mitoxantrone did not potentiate fluvastatin in either cell line and therefore was not further investigated. To determine the nature of the anti-proliferative activity of the statin-compound combinations, we evaluated cell death by two independent assays (fixed propidium iodide staining and PARP cleavage) with selumetinib, nelfinavir, or honokiol. Our data indicate that all three compounds, at sub-lethal concentrations, potentiate statin-induced cell death (Fig. 2).

**Fig. 3:**
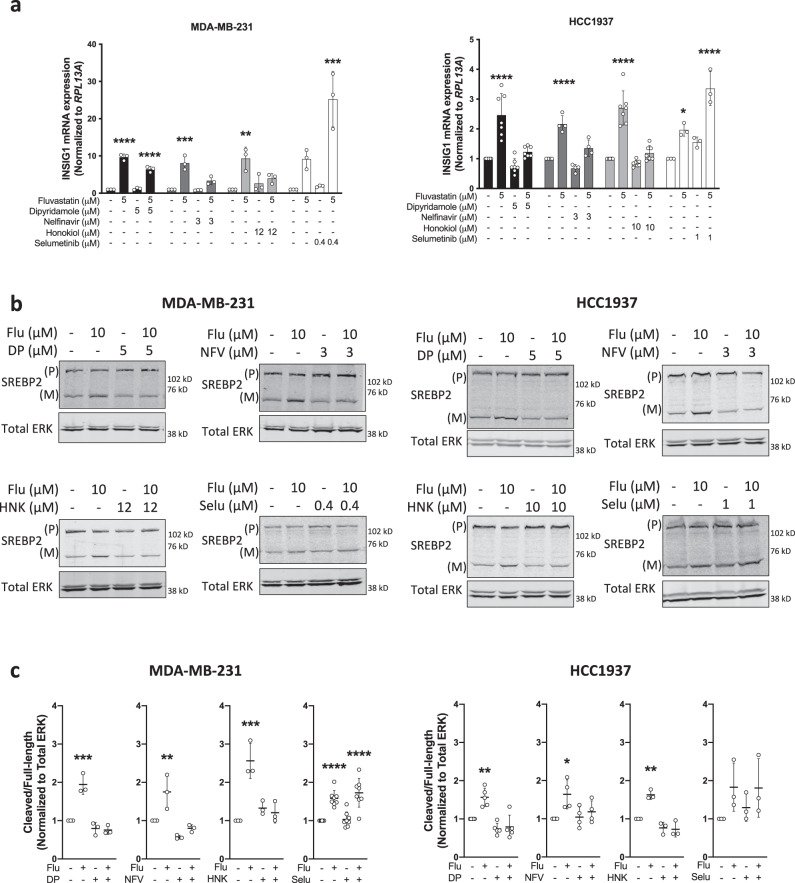
Nelfinavir and Honokiol block fluvastatin-induced SREBP2 activation. **a** MDA-MB-231 (left) and HCC1937 (right) cells were exposed to solvent controls, fluvastatin (Flu) +/− dipyridamole (DP), nelfinavir (NFV), honokiol (HNK) or selumetinib (Selu) for 16 h, and RNA was isolated to assay *INSIG1* expression by qRT-PCR. mRNA expression data are normalized to *RPL13A* expression. Error bars represent the mean ± SD, *n* = 3–4 biologically independent samples, *p < 0.05, **p < 0.01, ***p < 0.001, ****p < 0.0001 (one-way ANOVA with Bonferroni’s multiple comparisons test, where each group was compared to the solvent control group within each experiment). **b** MDA-MB-231 and HCC1937 cells were treated with fluvastatin + /− dipyridamole, nelfinavir, honokiol or selumetinib for 12 h, and protein was harvested to assay for SREBP2 expression and cleavage (activation) by immunoblotting. (P) represents precursor, full-length SREBP2, and (M) represents mature, cleaved SREBP2. Total ERK was assayed as a protein loading control. *N* = 3–8 biologically independent experiements, the representative image is shown. **c** SREBP2 cleavage (cleaved/full-length) was quantified by densitometry and normalized to total ERK expression. Error bars represent the mean + /− SD, *n* = 3–8 biologically independent samples, *p < 0.05, **p < 0.01, ***p < 0.001, ****p < 0.0001 (one-way ANOVA with Bonferroni’s multiple comparisons test, where each group was compared to the solvent controls group within its experiment). Source data are provided as source data file.

Mechanistically, statins induce a feedback response mediated by SREBP2 that has been shown to dampen cancer cell sensitivity to statin exposure. Moreover, blocking the SREBP2-mediated feedback response with dipyridamole enhances statin-induced cancer cell death^23,25^. We have shown that dipyridamole blocks the regulatory cleavage, and therefore activation, of SREBP2, decreasing mRNA expression of SREBP2 target genes. As expected, statin treatment in both the MDA-MB-231 and HCC1937 cell lines induced the expression of SREBP2 target genes *INSIG1*, *HMGCR,* and *HMGCS1* after 16 hours of treatment, which was blocked by co-treatment with dipyridamole (Fig. 3a, Supplementary Fig. 5a). Similarly, nelfinavir and honokiol, but not selumetinib, blocked the statin-induced expression of MVA pathway genes (Fig. 3a, Supplementary Fig. 5a). Because SREBP2 is synthesized as an inactive, full-length precursor that is processed to its active, mature, nuclear form upon proteolytic cleavage, we used western blot analysis to assess the protein levels of both full-length precursor and mature SREBP2. Nelfinavir and honokiol, but not selumetinib, blocked fluvastatin-induced SREBP2 processing and cleavage, similar to dipyridamole (Fig. 3b, c), which is consistent with inhibited SREBP2 transcriptional activity (Fig. 3a). This suggests that while selumetinib is a strong potentiator of statin-induced cell death, there is no evidence that selumetinib regulates SREBP2 activity suggesting another mode of potentiation (Fig. 3, Supplementary Fig. 5a). Thus MVA-DNF successfully identified nelfinavir and honokiol as potentiators of fluvastatin-induced cell death by blocking the SREBP2-mediated feedback response.

Dipyridamole is approved as a platelet aggregation inhibitor. Therefore, to investigate the anti-platelet activity of nelfinavir and honokiol, we evaluated the ability of these compounds to block thrombin receptor-activating protein 6 (TRAP-6)-induced platelet aggregation. Using whole blood obtained from healthy donors not on medication, aggregation induced by TRAP-6 was measured using a Multiplate Analyzer and compared to no inhibitor controls. Although aggregation was decreased by dipyridamole (DP), there was no effect of nelfinavir (NFV) or honokiol (HNK) (Supplementary Fig. 5b).

### Statin-compound combinations are synergistic in a breast cancer cell line screen

**Fig. 4:**
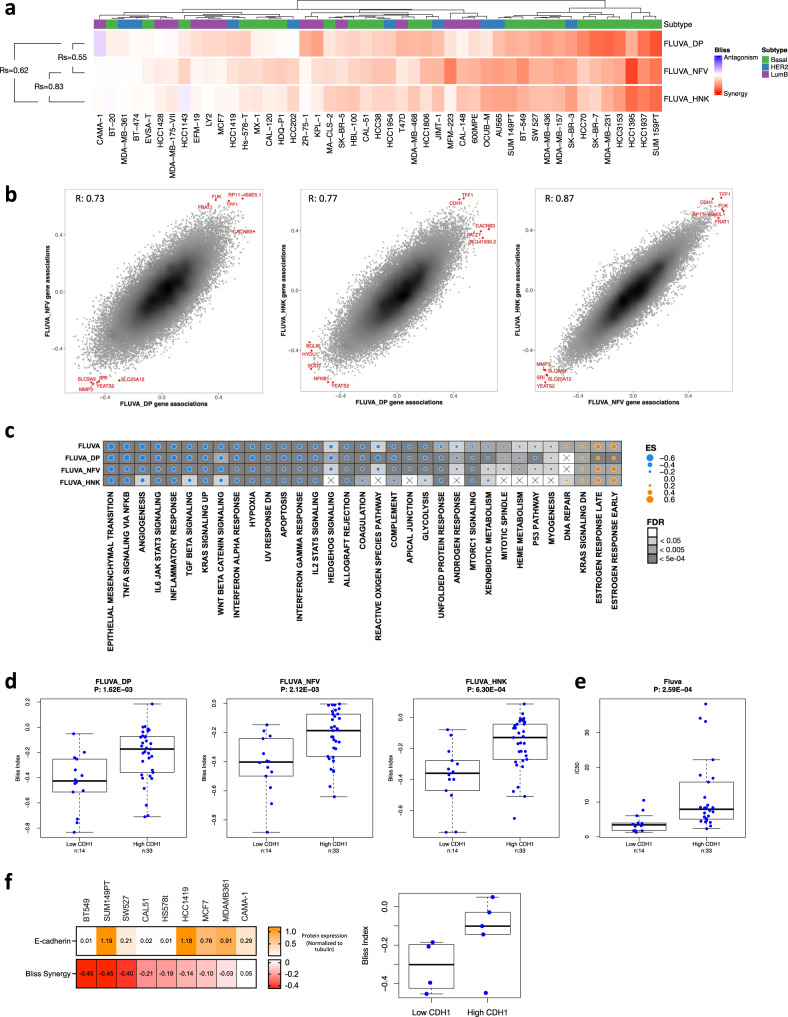
Compound combination synergy analysis identified basal E-cadherin to predict synergistic response to fluvastatin-compound combinations. **a** Heatmap of synergy scores (Bliss Index model) for fluvastatin (Fluva) + dipyridamole (DP), nelfinavir (NFV) or honokiol (HNK) in a panel of 47 BC cells lines. Clustered by synergy score from least (>0) to greatest (<0) synergy. BC subtype of each cell line shown is based on the SCMOD2 subtyping scheme. Spearman correlation coefficients are labeled on the left. **b** Basal mRNA expression^40^ associations with synergy scores for each drug combination (e.g. Fluva-NFV vs. Fluva-DP, Fluva-HNK vs. Fluva-DP, and Fluva-NFV vs. Fluva-HNK). Associations were calculated using Pearson correlation coefficient. Top five basally-expressed genes associated with synergy in either direction are annotated in red. **c** Gene set enrichment analysis (GSEA) using the Hallmark gene set collection, where genes were ranked according to their correlation to the fluvastatin IC_50_ (Fluva) value or to the synergy score (Fluva-DP, Fluva-NFV and Fluva-HNK). Dot plot was restricted to pathways enriched in at least two out of the four groups. Dot size indicates the difference in enrichment scores (ES) of the pathways. Background shading indicates FDR and X indicates pathway and drug combinations that were not significantly enriched (FDR > 0.05). **d** Basal E-cadherin mRNA expression between cell lines is predicted to be synergistic or not to the drug combination. Synergy was defined by Bliss Index and significance was measured by two-sided wilcoxon rank sum test. **e** Basal E-cadherin mRNA expression between cell lines predicted to be respondent or not to fluvastatin. Sensitivity was defined by IC_50_ and significance was measured by two-sided wilcoxon rank sum test. **f** BC cell line E-cadherin protein expression is inversely correlated with Bliss synergy. Densitometry values of E-cadherin expression normalized to tubulin plotted as a heatmap (orange is high E-cad protein expression). Average Bliss synergy score for the corresponding cell lines (red is high Bliss synergy score) (left). Average Bliss synergy score plotted by E-cadherin expression binarized based on z-score (right). **d**–**f** The center lines, bounds of box, whiskers, points of boxplot indicate median, lower/upper quartile (25th/75th percentile), minima/maxima, and raw data, respectively.

To investigate the broad applicability of nelfinavir and honokiol as potentiators of fluvastatin, and examine the determinants of synergy, we further evaluated these statin-compound combinations across a large panel of 47 BC cell lines. A 5-day cytotoxicity assay (sulforhodamine B assay; SRB) in a 6 × 10 dose matrix was used to assess fluvastatin-compound efficacy. As expected, dipyridamole treatment resulted in a dose-dependent increase in fluvastatin area above the curve (AAC) value (Supplementary Fig. 6a). Similarly, nelfinavir and honokiol treatment also resulted in a dose-dependent increase in fluvastatin AAC values (Supplementary Fig. 6a). Based on these data, we evaluated statin-compound synergy using the Bliss Index model derived using SynergyFinder^38^ across the panel of BC cell lines. Like the dose-dependent sensitivity data, we observed that the trend in synergy between fluvastatin-dipyridamole across the 47 BC cell lines was also seen with fluvastatin-nelfinavir and fluvastatin-honokiol (Fig. 4a).

Since we had previously identified that the basal subtype of BC cell lines are more sensitive to fluvastatin as a single agent^18^, we evaluated whether basal BC cell lines were similarly more sensitive to the fluvastatin-compound combinations. Classifying the BC cell lines into basal, HER2, and luminal B subtypes^39^, we determined that the synergy of the fluvastatin-compound combinations is independent of subtype (Supplementary Fig. 6b). This suggests these statin-compound combinations can be applied to multiple BC subtypes as therapeutic options.

Overall the synergy profiles across the three fluvastatin-compound combinations were significantly similar (Fluva-NFV vs Fluva-DP, R_s_ = 0.55, *p*-value = 7.1e-05; Fluva-HNK vs Fluva-DP, R_s_ = 0.62, *p*-value = 5.5e-06; Fluva-NFV vs Fluva-HNK, R_s_ = 0.83, *p*-value <2.2e-16) (Fig. 4a). To further interrogate the similarity between the statin-compound combinations, we evaluated the correlation of the RNA-seq and reverse phase protein array (RPPA) profiles of the 47 BC cell lines^40^ with their synergy scores for each of the statin-compound combinations. These represent the transcriptomic and proteomic state associations with synergy for each combination, respectively. We then evaluated the correlation between these associations across the different combinations (Fluva-DP vs Fluva-NFV; Fluva-DP vs Fluva-HNK; Fluva-NFV vs Fluva-HNK) (Fig. 4b) and identified a high positive correlation between the combinations on the basis of similar transcriptomic associations (Fluva-NFV vs Fluva-DP, R_s_ = 0.73, *p*-value <2.2e-16; Fluva-HNK vs Fluva-DP, R_s_ = 0.77, *p*-value <2.2e-16; Fluva-NFV vs Fluva-HNK, R_s_ = 0.87, *p*-value <2.2e-16). This high positive correlation was also seen between these combinations using proteomic (RPPA) and synergy data (Supplementary Fig. 6c) suggesting that similar pathways were associated with the synergistic response to the three statin-compound combinations.

To compare the overlap in pathways associated with sensitivity to fluvastatin alone and synergy between the fluvastatin-compound combinations, a Gene Set Enrichment Analysis (GSEA) using the Hallmark Gene Set Collection was performed^41^. These results showed that enriched pathways were highly similar amongst fluvastatin alone and the fluvastatin-compound combinations with one of the highest scoring enriched pathways being epithelial-mesenchymal transition (EMT) (Fig. 4c). Because of the low agreement amongst EMT gene sets (Supplementary Fig. 7a), we more thoroughly tested this finding by evaluating four additional EMT gene sets. Similar trends were observed between fluvastatin alone and the fluvastatin-compound combinations for each of the EMT gene sets (Supplementary Fig. 6d). As we and others have published that mesenchymal-enriched cancer cell lines are more sensitive to statin monotherapy^42,43^, these data suggest that fluvastatin is the primary driver of response to these statin-compound combinations. This is consistent with fluvastatin inhibiting the MVA pathway, triggering the SREBP2-mediated feedback response, which in turn is inhibited by the second compound (dipyridamole, nelfinavir, or honokiol) in these fluvastatin-compound combinations.

The individual genes within each of the EMT gene sets were examined to identify a biomarker of the synergistic response to the statin-compound combinations. As mentioned, within the EMT field, gene set signatures have low agreement (Supplementary Fig. 7a). A previously published binary classifier of five EMT genes could predict increased sensitivity to statins across 631 cell lines representing multiple cancer types^42^. This five-gene classifier was tested to see if it could also predict synergy between the different fluvastatin-compound combinations. As expected, the EMT classifier could predict sensitivity to fluvastatin alone across the panel of BC cell lines (Supplementary Fig. 8a) but failed to predict synergy to the fluvastatin-compound combinations (Supplementary Fig. 8b). To further evaluate EMT, each of the five genes were interrogated individually. Expressions of these genes were binarized based on z-score. Interestingly, low gene expression and protein levels of E-cadherin (*CDH1*), a canonical epithelial state marker, not only predicted sensitivity to fluvastatin alone but also drug synergy across all three fluvastatin-compound combinations (Fig. 4d, e and Supplementary Fig. 8c). To validate this finding, E-cadherin protein expression was probed for across a panel of nine BC cell lines and showed that synergy to the fluvastatin-compound combinations is positively associated with low E-cadherin protein expression in 8 out of the 9 BC cell lines (BT549, SW527, CAL51, HS578t, HCC1419, MCF7, CAMA-1, and MDAMB361) (Fig. 4f and Supplementary Fig. 7b).

### Fluvastatin and nelfinavir synergistically trigger cell death of breast cancer patient samples

**Fig. 5:**
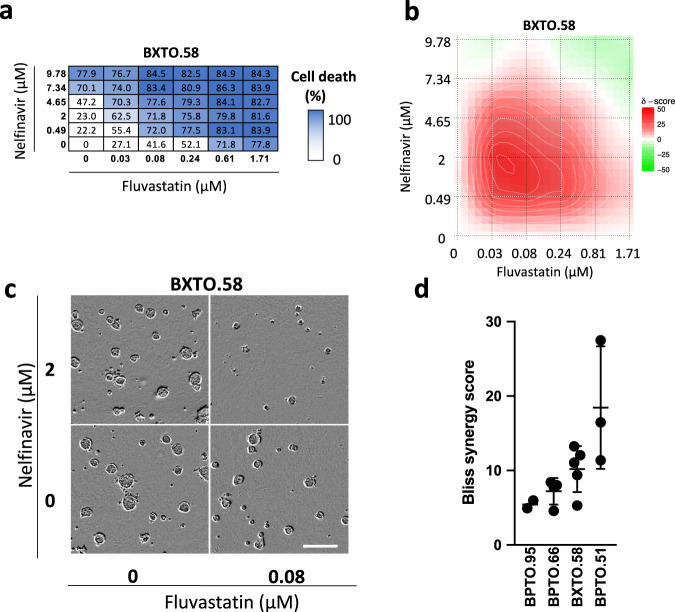
Fluvastatin-nelfinavir combination is synergistic in primary breast cancer patient-derived tumor organoids. **a** Cell death matrix for BXTO.58 organoids as determined by CellTiter-Glo. Each well was normalized to the DMSO control well (0% cell death). Visualizing the 20th to 80th percentile. **b** Synergy plot for BXTO.58 organoids treated with the indicated doses of fluvastatin and nelfinavir, where red represents synergy and green represents antagonism. **c** Representative images of BXTO.58 organoids are shown after 7 days of treatment. Selected images represent the most synergistic area identified through SynergyFinder. *N* = 2–5 biologically independent experiments. Scale bars = 200 μm. **d** The Bliss synergy scores for the four organoid models tested are plotted. The data are represented as the mean + /− SD, *n* = 2–5 biologically independent experiments.

To further evaluate the 2D cell culture findings, we developed a 3D primary BC patient-derived tumor organoid assay to evaluate fluvastatin-nelfinavir activity, alone and in combination. We prioritized nelfinavir to test in combination with fluvastatin over honokiol because it is a protease inhibitor with S2P activity that is FDA-approved, and has not been used in combination with statins as an anticancer agent. Single cells were seeded in basement membrane extract, treated with a concentration range of fluvastatin and/or nelfinavir, and allowed to form 3D structures. Cell viability was assessed through an ATP-based luminescence assay (Fig. 5a) and used to generate synergy scores across the dose matrix tested (Fig. 5b and Supplementary Fig. 9a). Live cell imaging was also used to monitor organoid growth (Fig. 5c and Supplementary Fig. 9b). We screened four patient-derived BC organoids with fluvastatin and nelfinavir (Fig. 5d). In agreement with the 2D cell death assays, the fluvastatin-nelfinavir combination synergised to impair organoid cell viability and growth in the BC patient-derived organoids.

### Additional validation of compounds identified through our pharmacogenomics approach

**Fig. 6:**
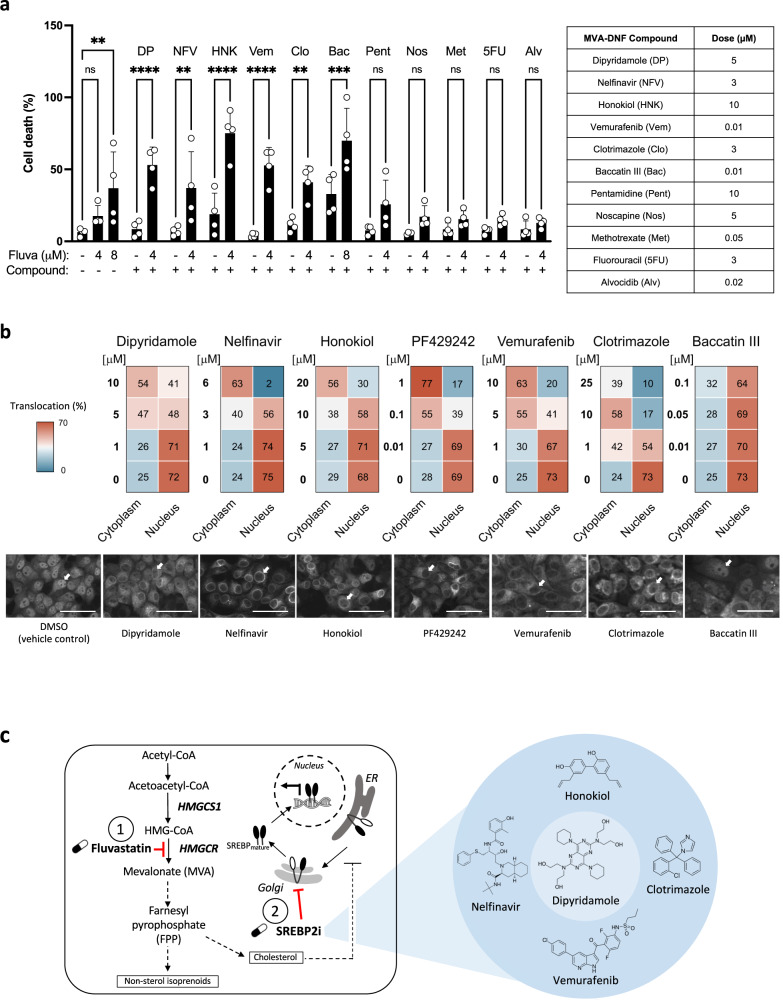
Cell death and SREBP2 translocation assays validate additional pharmacogenomic MVA-DNF compounds. **a** HCC1937 cells were treated with a concentration range of MVA-DNF compounds with and without 4 μM fluvastatin for 72 h. Cells were then stained with DRAQ5, TMRE and Caspase-3/7 and subsequently imaged by confocal microscopy. Quantification of percent cell death was determined from linear classification analysis. Error bars represent the mean + /− SD, *n* = 4 biologically independent experiments, *p < 0.05, **p < 0.01, ***p < 0.001, ****p < 0.0001 (one-way ANOVA with Bonferroni’s multiple comparisons test, where each group was compared to the indicated group). **b** Classification result of subcellular localization of mNeon-SREBP2 in NMuMG cells in the presence of fluvastatin (10 µM) and geranylgeranyl pyrophosphate (GGPP) (2 µM) treated with a set of compounds (high, medium, low dose) as indicated in the legend, identified to potentiate fluvastatin-induced cell death from **(a)** for 16 h. Treatment was carried out in 5% lipoprotein-deficient serum (LPDS)-supplemented culture media. Numbers shown within the heat map indicate the percentage of cells assigned either to the cytoplasm or nucleus. While RanGTP functions as a nuclear landmark, the Cytoplasm comprises multiple organelle markers: endoplasmic reticulum, Golgi apparatus, nuclear envelope, and secretory pathway. The shown result is representative of three replicates. Sample micrographs of mNeon-SREBP2 expressed in NMuMG cells treated with either fluvastatin/GGPP in the presence of the respective highest dose of dipyridamole, nelfinavir, honokiol, PF429242, vemurafenib, clotrimazole or baccatin III for 16 h. The white arrow indicates nuclei of interest. Scale bar = 50 μm. **c** Schematic diagram detailing the potential for fluvastatin (labeled with 1) and SREBP2 inhibitors (labeled with SREBP2i) to block the SREBP2-mediated feedback response and synergise to potentiate fluvastatin-induced cell death.

To interrogate the remaining compounds identified by MVA-DNF, we conducted two secondary assays. We prioritized an additional 8 of the 13 remaining MVA-DNF compounds based on clinical relevance in BC in addition to the negative control (DMSO), positive control (dipyridamole), and validated MVA-DNF compounds (nelfinavir and honokiol). First, we evaluated the ability of the compounds to potentiate fluvastatin and increase cell death using a live-cell imaging assay. We treated HCC1937 cells with a sublethal concentration range of fluvastatin, the compounds identified using MVA-DNF, or the combination for 72 h, after which cells were stained with DRAQ5 (DNA dye), TMRE (marker of active mitochondria) and Caspase 3/7 (marker of apoptotic cells**)** (Supplementary Fig 10a, b). The stained cells were then imaged by confocal microscopy and the images were subjected to linear classification analysis to determine the percentage of dead cells for each treatment (single agent and in combination with fluvastatin). Caspase 3/7 staining is not fully captured in the image as dying cells round up, and the signal is not in the same focal plane as the mitochondria. Each fluvastatin-MVA-DNF compound combination was compared to the single agent. We observed significant potentiation of fluvastatin with positive control dipyridamole, validated potentiators nelfinavir and honokiol, and three MVA-DNF compounds: vemurafenib (RAF/MEK inhibitor), clotrimazole (antifungal), and baccatin III (natural product) (Fig. 6a).

To further validate these results and determine mechanism of statin potentiation, we focused on the three MVA-DNF compounds (vemurafenib, clotrimazole and baccatin III) that potentiated fluvastatin-induced cell death and interrogated these compounds for their ability to block the SREBP2-mediated feedback response. We developed a mNeon-SREBP2 cytoplasmic-nuclear translocation assay in a non-transformed mouse mammary gland epithelial cell line (NMuMG). These cells were chosen as they have an intact sterol feedback response, and there is an established cell image reference library of organelle markers (landmarks) that can be used to automatically assign the subcellular localization of mNeon-SREBP2 by image-based machine learning^44^. As expected, exposure of the cells to lipoprotein deficient serum (LPDS) and either i) fluvastatin and geranylgeranyl pyrophosphate (GGPP), or ii) U18666A, resulted in nuclear translocation of mNeon-SREBP2. Fluvastatin inhibits HMGCR and GGPP blocks fluvastatin-induced cell death^45^, while U18666A is an inhibitor of cholesterol synthesis and transport (Fig. 6b and Supplementary Fig. 11a, b).

To identify inhibitors of SREBP2 translocation, we exposed the mNeon-SREBP2 NMuMG cells for 16 h with the agonists to drive SREBP2 activation (fluvastatin/GGPP or U18666A in LPDS) and a concentration range of positive control compounds that block the feedback response^46^ (dipyridamole, nelfinavir, honokiol) and PF429242 (site-1 protease inhibitor) as well as the additional MVA-DNF compound hits that potentiated statin-induced cell death (vemurafenib, clotrimazole and baccatin III). The treated cells were imaged by confocal microscopy and subcellular localization was assigned as either nuclear or cytoplasmic using the classifier associated with the organelle reference library^44^ (Fig. 6b). As expected, the positive controls dipyridamole and PF429242 blocked fluvastatin/GGPP induced mNeon-SREBP2 nuclear translocation 72% to 41% and 69% to <20%, respectively. Moreover, mNeon-SREBP2 nuclear translocation was blocked by nelfinavir (75% to <20%) and honokiol (68% to 30%) (Fig. 6b) consistent with the results presented above (Fig. 3b). Similar dose-dependent inhibition of nuclear translocation was evident when U18666A was used as the translocation agonist (Supp Fig 11a, b), consistent with the translocation results seen by immunoblotting **(**Fig. 3b). Translocation of mNeon-SREBP2 was also blocked by vemurafenib (73% to 20%) and clotrimazole (73% to <20%), but not by baccatin III (73% to 64%). These data suggest that both vemurafenib and clotrimazole potentiate fluvastatin induced cell death by blocking the SREBP2-mediated feedback response, whereas baccatin III potentiates fluvastatin via an alternative mechanism.

## Discussion

Dipyridamole potentiates statin efficacy to drive tumor cell death by blocking the statin-induced restorative feedback response of the MVA pathway^23,25^. Statins are readily available, safe, approved and manufactured as inexpensive generic drugs. Our goal was to expand the number of agents that potentiate the pro-apoptotic activity of statins to ultimately better use statins as anticancer agents at clinically achievable doses. Systematic and targeted efforts have been made in the past to identify drugs that potentiate statins’ anticancer effects^47–52^. To this end, we used a computational pharmacogenomics pipeline to enrich for compounds with similar properties to our prototypic compound, dipyridamole, at the level of structure, anti-proliferative activity and MVA pathway gene expression perturbation. We identified 19 potential compounds and evaluated several for their ability to potentiate statin-induced apoptosis by blocking the restorative feedback loop of the MVA pathway. By this approach, we first validated nelfinavir and honokiol from the prioritized hits by combining fluvastatin with each of these agents, whereby the concentration of fluvastatin needed to induce cell death was then lowered to a clinically-achievable range^53,54^. Analysis of basal RNA and protein expression identified the epithelial cell marker, *CDH1* (E-cadherin) as a biomarker of the synergistic response to both fluvastatin-nelfinavir and fluvastatin-honokiol treatment. From the remaining hits, we identified another 2 compounds, clotrimazole and vemurafenib, that potentiate fluvastatin-induced cell death and block SREBP2 activity.

In addition to blocking statin-induced SREBP2 activity, several other activities of dipyridamole have been described, including inhibition of phosphodiesterases^55^, nucleoside transport^56^ and glucose uptake^57^. By restricting the gene perturbation layer of the pharmacogenomics pipeline to MVA pathway genes, our rationale was to bypass these extraneous activities and focus our analysis on identifying drugs whose mechanisms centered on the MVA pathway. This highlights that the computational pharmacogenomics pipeline described here is likely tunable to drug-specific structural features, activities and signaling pathways. Indeed, as the two pharmacogenomic data sets used here continue to increase in size, these additional drugs and genes can be leveraged to further customize the analysis.

The first two statin-sensitizing agents identified using MVA-DNF include nelfinavir and honokiol, which we demonstrate here inhibit statin-induced SREBP2 cleavage and activation similar to dipyridamole^23,25^. To date, a number of agents have been identified that block SREBP2 activation, including fatostatin, betulin, and xanthohumal, which block ER-Golgi translocation. Additional SREBP2 inhibitors include BF175 and tocotrienols that target SREBP2 transcriptional activity and protein stability, respectively. However, other than nelfinavir, these agents are either under development for clinical application or are only used for research purposes and are not likely to be advanced to patient care. The S2P protease inhibitor nelfinavir (marketed as Viracept) was approved for use in 1997 as an antiviral for the treatment of HIV, and is under evaluation for its utility as an anticancer agent^58–63^. This further reinforces that the combination of statin-nelfinavir is immediately actionable and should be further evaluated at the clinical level without delay. We suggest the fluvastatin-nelfinavir combination is preferred compared to other statins, as distinct cytochrome P450 enzymes are used to process these agents, thereby reducing the potential for adverse drug-drug interactions^64^.

To our knowledge, honokiol in combination with statins in the context of cancer has not been well investigated. Honokiol is a natural product commonly used in traditional medicine and has a number of reported mechanisms of action. Here we show that honokiol inhibits SREBP2 translocation and induction of gene expression in combination with fluvastatin. As honokiol and its derivatives are presently under investigation, discovering this activity for honokiol can be incorporated into future analyses of structure-activity relationships of this agent.

We also observed this fluvastatin-nelfinavir and fluvastatin-honokiol synergistic response to the combination therapies across multiple subtypes of BC. Previously, we and others had identified the basal-like BC subtype as more sensitive to statins alone and identified a mesenchymal-enriched gene expression profile as highly predictive of statin sensitivity^42,65,66^. Here, we have expanded the scope of statin treatment to encompass a wider spectrum of BCs when used as combination therapy. Moreover, analyses of gene and protein expression data across a large collection of BC cell lines identified canonical epithelial cell marker *CDH1* as predictive of synergy to all three statin-compound (dipyridamole, nelfinavir or honokiol) combinations. We further showed that low *CDH1* expression levels served as a biomarker of synergistic response in BC cell lines^67^. Other groups have independently correlated E-cadherin expression with statin resistance and suggested its use as a biomarker of statin sensitivity^67^. This suggests further evaluation of *CDH1* as a biomarker is warranted in addition to previously published gene expression signatures for statin sensitivity^48^.

To expand upon the 2D cell culture findings, we screened four 3D primary BC patient-derived tumor organoids to evaluate fluvastatin-nelfinavir activity, alone and in combination. All four patient-derived organoid models were synergistic to fluvastatin-nelfinavir. Synergy was observed at physiologically achievable^23,45,53^ concentrations of fluvastatin and low nanomolar concentrations of nelfinavir.

To further validate the remaining MVA-DNF compounds, we used two high-throughput screening assays to identify compounds that could first potentiate fluvastatin-induced cell death and second block the SREBP2 mediated feedback response. First, we leveraged a live-cell imaging assay that integrates the results from three independent live-cell dyes to determine cell death. Second, we developed a live-cell imaging assay to quantify SREBP2 cytoplasmic-to-nuclear translocation. Using this approach, we identified two additional SREBP2 inhibitors, clotrimazole and vemurafenib, as statin-sensitizers. Clotrimazole is a topical antifungal agent and vemurafenib is an oral V600E BRAF inhibitor. The mechanism of both these compounds as SREBP2 inhibitors remains unclear and warrants further investigation.

Additional compounds tested in this study include selumetinib, baccatin III and mitoxantrone; the former two were observed to sensitize BC cells to statin-induced apoptosis, but the latter did not. Our data suggest that selumetinib and baccatin III function through a SREBP2-independent mechanism. Drugs that function through alternative mechanisms of statin potentiation identified using our method were anticipated due to the selection criteria in the MVA-DNF pipeline. The identification of such compounds is potentially advantageous as some multiple myeloma and prostate cancer cell lines have been shown to lack statin-induced SREBP2 activity^22,23,45^. Future investigation to assess the effectiveness of the drug combinations in ex-vivo and murine models of primary tumor growth and metastasis is warranted. Testing of drug combinations using additional models can provide insights that are currently lacking from testing drug combinations in vitro. These include the tumor microenvironment and immune cell interactions.

The data presented here have important clinical implications for statins as anticancer agents. Despite encouraging results from window-of-opportunity clinical trials in BC using statins as a single-agent, only a modest effect was observed with some but not all patients^20,21^. Accordingly, discovery of therapeutic combinations is necessary to achieve significant clinical impact. Our study provides a strong preclinical rationale to warrant further investigation of the fluvastatin-nelfinavir, fluvastatin-honokiol, fluvastatin-clotrimazole and fluvastatin-vemurafenib combinations, as well as the utility of *CDH1* as a biomarker of response. Since nelfinavir and vemurafenib are poised for repurposing, and statins have demonstrated anticancer activity in early-phase clinical trials^20,21,53,68–72^, clinical studies to further evaluate the therapeutic benefit of these combinations can proceed swiftly. We validated the pharmacogenomic pipeline using breast cancer as a model system, however these statin-compound treatment options may also be effective for additional cancers in which the mevalonate pathway is contributing to disease. The availability of these approved, well-tolerated, oral drugs as well as simple methods for assessing *CDH1* expression could enable rapid translation of these findings to improve cancer patient outcomes.

## Methods

This study was performed in accordance with the University Health Network (UHN) Research Ethics Board protocols (14-8358).

### MVA-specific Drug Network Fusion (MVA-DNF)

We developed a computational pharmacogenomic pipeline (MVA-DNF) that facilitates the identification of compounds that block the SREBP mediated feedback response and potentiate fluvastatin-induced cell death, by elucidating drug-drug relationships specific to the mevalonate (MVA) pathway. This MVA-DNF pipeline extends on some principles of our previously described drug network fusion algorithm^29^, by utilizing the similarity network fusion algorithm across three drug taxonomies. The MVA-DNF pipeline expands on core principles which include evaluation of your “reference” compound at three levels: drug structure, drug sensitivity, and drug perturbation. Drug structure annotations and drug perturbation signatures are obtained from the LINCS-L1000 dataset^30^, and drug sensitivity signatures are obtained from the NCI-60 drug panel^31^. Drug structure annotations were converted into drug similarity matrices by calculating tanimoto similarity measures^73^ and extended connectivity fingerprints^74^ across all compounds, as described^29^. We extracted calculated Z-scores from drug-dose response curves for the NCI-60 drug sensitivity profiles and computed Pearson correlation across these profiles to generate a drug similarity matrix based on sensitivity^31^. We used our PharmacoGx package (version 1.6.1) to compute drug perturbation signatures for the L1000 dataset using a linear regression model^75^. The regression model adjusts for cell specific differences, batch effects, and experiment duration, to generate a signature for the effect of drug concentration on the transcriptional state of a cell. This facilitates the identification of gene expression which has been significantly perturbed due to drug treatment. These signatures indicate transcriptional changes that are induced by compounds on cancer cell lines. We further refined the drug perturbation profiles to a set of six MVA-pathway genes (Supplementary Fig 1a) that had been obtained from the literature as well as repositories of pathway-specific gene sets, including MSigDB^76^, HumanCyc^77^, and KEGG^78,79^. These gene sets include ‘mevalonate pathway’ and ‘superpathway of geranylgeranyldiphosphate biosynthesis I (via mevalonate)’ from the HumanCyc^80^, and ‘Kegg Terpenoid Backbone Biosynthesis’ from KEGG^78,79^. The filtered drug-induced gene perturbation signatures were subsequently used to generate a drug perturbation similarity matrix that elucidates drug-drug relationships based on common transcriptional changes across the six MVA-pathway genes. We calculated the similarity between estimated standardized coefficients of drug perturbation signatures using the Pearson correlation coefficient. Finally, we used the similarity network fusion algorithm^32^ to integrate the affinity matrices for drug structure, drug sensitivity, and MVA-pathway specific drug perturbation profiles, to generate an MVA-pathway specific drug taxonomy (MVA-DNF) spanning 238 compounds.

### Identification of compounds

We interrogated the MVA-DNF taxonomy using a variety of approaches to identify a set of candidate compounds. Using MVA-DNF similarity scores, we first generated a ranking of all compounds closest to dipyridamole, our reference input. We then conducted a permutation test, to assess the statistical relationship of each ranked drug against dipyridamole. Briefly, drug fusion networks were generated 999 times across perturbation, sensitivity, and drug structure profiles, each time using a random set of six genes to generate a ‘pathway-centric’ drug perturbation similarity matrix. Z-scores were calculated to determine the statistical relevance of a given compound in MVA-DNF, compared to the randomly generated networks. Corresponding p-values for each drug were calculated using the cumulative distribution function (*pnorm* with the default lower.tail=TRUE) from the R stats package. From this, we further ranked a list of candidate compounds by their statistical significance within MVA-DNF (*p*-value < 0.05) and Z-score < −1.8, resulting in the identification of 19 candidate compounds.

For each of the compounds we identified, we conducted a similar assessment of significance to identify the relationships of these compounds to dipyridamole and to themselves. A drug network was rendered using the iGraph R package^81^. We assessed the regulation of gene expression for genes involved in the mevalonate pathway across all of the top-selected compounds, by analyzing the drug-induced transcriptional profiles (described above). Using MVA-DNF similarity scores, we further assessed the contribution of each of the drug layers (structure, sensitivity and perturbation) towards the identification of these compounds. A radar plot was generated that compared the score between DP and each of the compounds identified, using the affinity matrices pertaining to the structure, sensitivity, and perturbation layers. To further prioritize the hits, candidate compounds were categorized by clinical relevance and known mechanism of action. Compounds that were known toxins or carcinogens were excluded from the analysis (Supplementary Table 1, Supplementary Fig 1b). The top five hits were selected for initial validation.

We interrogated whether using some of our top hits as the reference input would converge to the same set of drug hits within our MVA-centric pipeline. We retested the MVA-DNF pipeline using the same set of 6 MVA genes and using each of nelfinavir and honokiol as our prototype drugs in place of dipyridamole. MVA-DNF and permutation testing was conducted to identify candidate compounds that rank closest to nelfinavir or honokiol using *p <* 0.05 and z-score < −1.8. Overlaps between candidate compound hits were computed using the UpsetR^82^ package in R.

While our MVA-DNF is focused on an MVA-specific drug perturbation layer composed of six genes, we additionally tested the robustness and stability of generating MVA-DNF using an increasing number of genes as part of our drug perturbation layer. We tested the overall speed and progress of the MVA-DNF and permutation testing pipeline, as a function of time spent processing the data (CPU time in seconds that the overall pipeline has taken) and computer memory consumed (in megabytes) when running the pipeline on different gene sizes. We re-ran the MVA-DNF pipeline using a different number of random genes in the drug perturbation layer (n = 6, 10, 100, 500, 900 genes), and then running 999 random permutations of the MVA-DNF for each of the designated gene sizes as part of the permutation testing pipeline. We computed the runtime for one complete run of the pipeline (ie, 1000 runs of MVA-DNF) using the *proc.time()* function in R, and computed overall megabytes of RAM consumed using the *mem_used()* function of the pryr package^83^ in R. For each of the gene sizes tested, we conducted 5 complete runs of the pipeline to assess overall performance over multiple runs of MVA-DNF.

### Cell culture and compounds

All cell lines were cultured as described previously^18,25^. Briefly, MDA-MB-231 and HCC1937 cells were a gift from Mona Gauthier and cultured in Dulbecco’s Modified Eagle’s Medium (DMEM) and Roswell Park Memorial Institute medium (RPMI), respectively. Normal murine mammary gland (NMuMG) cells were a gift of J. Wrana, Lunenfeld-Tanenbaum Research Institute, Toronto, Canada, and cultured in DMEM (Gibco), containing 10 μg/ml bovine insulin (Sigma). HEK293T were a gift from Frank Graham at McMaster University, Hamilton, Canada, and grown in DMEM^84^. All cell lines were maintained in a 5% CO_2_ atmosphere at 37 °C. All media was supplemented with 10% fetal bovine serum (FBS), 100 units/mL penicillin and 100 μg/mL streptomycin. Cell lines were routinely confirmed to be mycoplasma-free using the MycoAlert Mycoplasma Detection Kit (Lonza), and their authenticity was verified by short-tandem repeat (STR) profiling at The Centre for Applied Genomics (Toronto, ON, Canada) Table 1.

**Table 1:** List of compounds used in manuscript

Compound	Company	Dissolved in
Fluvastatin (F5277-76)	US Biological	Ethanol or DMSO
Dipyridamole (D9766)	Sigma	DMSO
Honokiol (H4914)	Sigma	DMSO
Nelfinavir (PZ0013)	Sigma	DMSO
Mitoxantrone (M6545)	Sigma	DMSO
Selumetinib (S1008)	SelleckChem	DMSO
Clotrimazole (C6019)	Sigma	DMSO
Vemurafenib (PLX4032)	SelleckChem	DMSO
Baccatin III (Cephalomannine) (S2408)	SelleckChem	DMSO
Noscapine (HY-13716)	MedChem Express	DMSO
Methotrexate (A6770)	Sigma	DMSO
Pentamidine (S4007)	SelleckChem	DMSO
Fluorouracil (F6627)	Sigma	DMSO
Alvocidib (S1230)	SelleckChem	DMSO
PF429242 (15140)	Cayman Chemical	DMSO
Lipoprotein deficient serum (S5394)	Sigma	

### Breast cancer cell lines panel

The BC cell line^40^ panel was a generous gift from Dr. Benjamin Neel (Department of Medicine at NYU Grossman School of Medicine). RNAseq quantification was done using Kallisto pipeline^85^ using human transcriptome reference hg38.gencodeV23^86^. RPPA processed data was downloaded from Marcotte et al. 2016^40^. SCMOD2^87^ BC subtypes of these cell lines were obtained using the genefu R package^88^.

### Breast cell line combination viability screen

We used the sulforhodamine B colorimetric (SRB) proliferation assay^89^ in 96-well plates to determine drug dose-response curves. To test the fluvastatin-dipyridamole, fluvastatin-nelfinavir, and fluvastatin-honokiol drug combinations across the panel of BC cell lines (See Breast cancer cell lines panel), a 6 × 10 dose matrix format was used, covering a range of decreasing concentrations of each drug (highest drug dose was 20 μM fluvastatin, 20 μM dipyridamole, 10 μM nelfinavir, and 20 μM honokiol), along with all their pairwise combinations, as well as the negative controls (EtOH and DMSO). We subtracted the average phosphate-buffer saline (PBS) wells value from all wells and computed the standard deviation and coefficient for each replicate. All individually treated well values were normalized to the control well values. We used Prism (v8.2.0, GraphPad Software) to compute dose-response curves.

### Cell viability assays

Initial drug dose response curves were 3-(4,5-dimethylthiazol-2-yl)−2,5-diphenyltetrazolium bromide (MTT) assays were performed as previously described^22^ or imaged using the IncuCyte, an automated live-cell imaging instrument. Briefly, BC cells were seeded in 750-15,000 cells/well in 96-well plates overnight, then treated in triplicate with a dose range of selumetinib, nelfinavir, mitoxantrone or honokiol for single agent assays or fluvastatin at a sublethal compound dose. Half-maximal inhibitory concentrations (IC_50_) values were computed from dose-response curves using Prism (v8.2.0, GraphPad Software).

### Cell death assays

Cells were seeded at 2.5 × 10^5^ cells/plates and treated the next day as indicated. After 72 h, cells were fixed in 70% ethanol for >24 h, stained with propidium iodide and analyzed by flow cytometry for the sub-diploid (% pre-G1) DNA population as a measure of cell death as previously described^22^. Analysis was performed on a BD LSR II flow cytometer using BD FACSDIVA v8 and FlowJo v10 Software. The gating strategy used is provided in the Source Data file.

### Immunoblotting

Cell lysates were prepared by washing cells twice with cold PBS and lysing cells in RIPA buffer (50 mM Tris-HCl pH 8.0, 150 mM NaCl, 0.5% sodium deoxycholate, 1% NP-40, 0.1% SDS, 1 mM EDTA, protease inhibitors) on ice for 30 min. Lysates were cleared by centrifugation and protein concentrations were determined using the Pierce 660 nm Protein Assay Kit (Thermo Fisher Scientific). Equal amounts of protein were diluted in Laemmli sample buffer, boiled for 5 min and resolved by SDS-polyacrylamide gel electrophoresis. The resolved proteins were then transferred onto nitrocellulose membranes. Membranes were then blocked for 1 h in 5% milk in tris-buffered saline/0.1 % Tween-20 (TBS-T) at room temperature, then probed with the following primary antibodies in 5% milk/TBS-T overnight at 4 °C: SREBP-2 (1:250, BD Biosciences, 557037), p44/42 MAPK (ERK1/2) (1:1000, Cell Signaling Technology, 4695), PARP (1:1000, Cell Signaling Technology, 9542 L), ɑ-Tubulin (1:3000, Calbiochem, CP06) and E-cadherin (1:1000, Cell Signaling Technology, 3195). Primary antibodies were detected using IRDye-conjugated secondary antibodies (1:20,000, LI-COR Biosciences, 926-32211 and 926-32210) and the Odyssey Classic Imaging System (LI-COR Biosciences). Densitometric analysis was performed using ImageJ v1.47 software.

### RNA expression analyses

Total RNA was harvested from sub-confluent cells using TRIzol Reagent (Invitrogen). cDNA was synthesized from 500 ng RNA using SuperScript III (Invitrogen). Quantitative reverse transcription PCR (qRT-PCR) was performed using the ABI Prism 7900HT sequence detection system and TaqMan probes (Applied Biosystems) for *HMGCR* (Hs00168352), *HMGCS1* (Hs00266810), *INSIG1* (Hs01650979) and *RPL13A* (Hs01578913).

### Anti-platelet analysis

Whole blood was collected into 3.2% sodium citrate (Becton Dickinson) at a ratio of 9:1 (vol:vol) from healthy donors who provided written consent and were compensated financially (REB: Hamilton Integrated Research Ethics Board #4804). Whole blood (300 μL) was mixed with an equal volume of normal saline containing 3 mmol/L CaCl_2_ in the cup portion of a Multiplate test cell (Roche). Dipyridamole (10 µM), nelfinavir (10 µM) or honokiol (10 µM) or normal saline containing 3 mmol/L CaCl_2_ diluent (66 µL) were added. After a three-minute incubation at 37 °C, platelet activation in whole blood was initiated by adding 32 μmol/L thrombin receptor-activating protein 6 (TRAP-6) (Bachem, 4017752). Electrical impedance was recorded for 10 min using a Multiplate Analyzer (Roche). Values were expressed as aggregation, normalized to TRAP-6 alone in that donor.

### Drug combinations synergy analysis

Viability scores were calculated using standard pipelines from PharmacoGx R package^75^, and synergy scores represented by Bliss Index were calculated using SynergyFinder R package^38^. Pearson correlation coefficient was used to measure the associations between the transcriptomic and proteomic states of cell lines and the corresponding synergy scores for each of the combinations. The transcriptomic associations were then used to rank genes for GSEA^90^. The Hallmark gene set collection^41^ was downloaded from MSigDB^91^. The Piano R package was used to run GSEA analysis^92^. Other EMT-related pathways, namely “GO Positive Regulation of Epithelial To Mesenchymal Transition”^93^, “GO Epithelial To Mesenchymal Transition”^93^, “SARRIO Epithelial-Mesenchymal Transition DN”^94^, and “SARRIO Epithelial-Mesenchymal Transition Up”^94^, were also downloaded from MSigDB for analysis. Expressions of these genes were binarized based on z-score.

### Live cell imaging to interrogate additional MVA-DNF compounds

HCC1937 cells were seeded at approximately ­­800 cells/well (16,000 cells/mL) in a 384-well imaging plate (PerkinElmer Cell Carrier Ultra, 6057300). Twenty-four hours later the cells were treated with indicated concentrations of the compounds or EtOH/DMSO (as a negative control) either alone or in combination for 72 h. Thirty minutes prior to imaging, cells were co-stained with 5 µM DRAQ5 (Biostatus, DR51000), 10 µM TMRE (Life Technologies, T669) and 5 µM IncuCyte® Caspase-3/7 Dye for Apoptosis (Sartorius, 4440). DRAQ5 staining was used to identify and segment individual cells as well as to measure nuclear condensation, TMRE was used to visualize loss of mitochondrial membrane potential and IncuCyte® Caspase-3/7 Dye detects activation of two caspases associated with ongoing apoptosis. Cells were imaged using an Opera Phenix automated confocal microscope (PerkinElmer) with a 20x air objective. Three channels were captured: (i) DRAQ5 (excitation laser: 640 nm; emission filter: 650–760 nm); (ii) TMRE (excitation laser: 561 nm; emission filter: 570–630 nm) and iii) Caspase-3/7 (excitation laser: 488 nm; emission filter: 500–550). It should be noted that the Caspase-3/7 staining in the well is not fully captured in the images shown because the change in morphology of the dying cells results in much of the signal not being in the same focal plane as the mitochondria. Three technical replicates were performed for each of three independent experiments (*n* = 3). Technical replicates were in separate wells, and a minimum of five different fields of view were acquired for each well. Image acquisition, calculation of intensity features for each channel, and image analysis were performed using Harmony software (PerkinElmer). Image sets were then subjected to linear classification analysis (PhenoLOGIC™) to determine “% Dead” cells for each treatment condition. Training sets consisting of approximately twenty cells per population (“healthy” or “dead”) were generated and morphological properties and intensity values were automatically calculated for every cell. PhenoLOGIC™ was then used to automatically select parameters that discriminate between “growing” cells and “dead” cells and subsequently a value for “% Dead” was calculated.

### SREBP2 translocation assay to interrogate MVA-DNF compounds

To generate mNeon-SREBP2 fluorescent fusion protein, the coding region of the *SREBF2* gene was amplified by PCR and cloned into the lentiviral vector pLVX-EF1a-mNeonGreen-IRES-Neomycin using NotI and SpeI restriction sites. To generate fluorescently-tagged RanGTP as a nuclear marker, RanGTP was fused to mScarlet-I in pLVX-EF1a-mScarlet-I-IRES-Puromycin by using BamHI and SpeI. To express stably SREBP2 and RanGTP in NMuMG cells the lentiviral DNA plasmid and both pPAX2 and pMD2.G plasmids were transfected into HEK293T cells at 1:1:0.1 ratios by means of polyethylenimine (PEI). After 72 h, the virus-containing supernatant was collected from the HEK293T cells and filtered through a 0.45-μm filter unit and transferred onto NMuMG cells. To obtain stable cell lines, selection was performed by adding either by G418 (Wisent) or puromycin (Sigma) 48 h after transduction.

NMuMG cells co-expressing mNeon-SREBP2 and mScarlet-I-RanGTP were seeded (three wells per drug treatment) in a 384-well microplate (CellCarrier-384 ultra, B128 SRI/160; Perkin Elmer) and allowed to grow for 24 h. After drug treatment (16hrs), plates were imaged (13 fields of view) on a spinning disk automated confocal microscope (OPERA Phenix®; PerkinElmer) with 40x water objective (NA = 0.9) in a defined temperature (37 °C) and CO_2_ (5%) environment. Images were collected using sCMOS cameras (4.6 MP, 16-bit), unbinned. Segmentation and feature extraction were carried out in CellProfiler software version 4.1.3.^95^. As described in Schormann et al.^44^ subcellular localization was determined by classifying the cell images using a Random Forests algorithm. The algorithm automatically identifies the main subcellular localization of the target proteins in each cell. For simplicity here the subcellular localizations were reduced to nuclear or cytoplasmic. The cytoplasmic designation includes localization in the Endoplasmic reticulum, secretory pathway and Golgi apparatus. Only cell image classifications above 20% are considered significant and shown in the results. Classification experiments were carried out in three replicates.

### Organoid synergy assay

Clear-bottom 384-well plates (Corning, 3712) were coated with a 10% matrix (3:1 ratio of Collagen I (PureCol Solution, 3 mg/mL, Advanced Biomatrix, 5005):Cultrex PathClear Reduced Growth Factor BME (Bio-Techne; 3533-010-02)) in breast organoid media^96^. Organoids were dissociated and seeded as single cells (750 cells/well) into previously prepared plates in breast organoid media containing 2% BME and treated the next day. To test the combinations in the panel of BC organoids, the fluvastatin/nelfinavir, drug combination was tested in a 6 × 6 dose matrix format covering a range of decreasing concentrations of each drug (the highest drug dose was 1.71 μM fluvastatin and 9.78 μM nelfinavir), along with all pairwise combinations, as well as the negative control (DMSO). Organoids were treated using the automated Tecan drug dispenser and imaged using the IncuCyte, an automated live-cell imaging assay. After 7 − 14 days of treatment, cell death was measured using Cell Titre-Glo 3D assay (Promega, 9682) and read using a CLARIOstar plate reader (BMG Labtech). All individually treated well values were normalized to the control well values. Dose-response matrices were then used to compute synergy scores using SynergyFinder^38^. All organoid models described in this study were generated from patient tissue obtained with informed consent and with UHN institutional REB approval (#14-8358; #17-5518 and #06-0196); the organoids were characterized and deposited with the Princess Margaret Living Biobank (www.livingbiobank.ca). No patient compensation was provided.

### Reporting summary

Further information on research design is available in the Nature Research Reporting Summary linked to this article.

## Supplementary information

Supplementary Information

Description of additional supplementary files

Supplemental table 1

Reporting Summary

## Data Availability

The reference subcellular image library data was uploaded to the Image Data Resource (idr0072; [https://idr.openmicroscopy.org/webclient/?show=screen-2952]). The data underlying Fig. 2, Fig. 3, Fig. 5, Fig. 6, Supplementary Figs. 7, 9, and 10 generated in this study are provided in the Source Data file. Raw data used to generate Fig. 1, Fig. 4, Supplementary Figs. 1 and 6–8, are publicly available on Github [https://github.com/DGendoo/MVA_DNF]. The LINCS-L1000 dataset containing profiles of drug-treated cancer cell lines can be downloaded from NCBI GEO (GSE70138 and GSE92742, which contains Level2 data for epsilon probes/features that represent raw gene expression/GEX, rendered as a GCTX file). NCI-60 compound sensitivity data (with average z-score) can be downloaded as ‘DTP_NCI60_ZSCORE.xlsx’ from the Cellminer website [https://discover.nci.nih.gov/cellminer/loadDownload.do] and selecting for ‘Compound activity: DTP NCI-60’. RPPA data is available from [http://neellab.github.io/bfg] and RNAseq data is available from Orcestra portal [https://www.orcestra.ca/pset/10.5281/zenodo.3905460]. Gene set collections for GSEA analysis can be downloaded from MSigDB [http://www.gsea-msigdb.org/gsea/msigdb/index.jsp]. Processed versions of these data are made available through our public GitHub repository [https://github.com/DGendoo/MVA_DNF]. Source data are provided with this paper.

## Source data

Source data file

## Code availability

The code and associated tutorial describing how to run the analysis pipeline are publicly available on Github (https://github.com/DGendoo/MVA_DNF). All software dependencies are available on Bioconductor (BioC) or the Comprehensive Repository R Archive Network (CRAN), and have been listed throughout the methods as applicable.

## References

[1] Canadian Cancer Society. Canadian Cancer Statistics publication. https://cdn.cancer.ca/-/media/files/research/cancer-statistics/2019-statistics/canadian-cancer-statistics-2019-en.pdf (2019).

[2] Lebert JM, Lester R, Powell E, Seal M, McCarthy J (2018). Advances in the systemic treatment of triple-negative breast cancer. Curr. Oncol..

[3] DeBerardinis RJ, Chandel NS (2016). Fundamentals of cancer metabolism. Sci. Adv..

[4] Warburg O (1956). On the origin of cancer cells. Science.

[5] Ehmsen S (2019). Increased cholesterol biosynthesis is a key characteristic of breast cancer stem cells influencing patient outcome. Cell Rep..

[6] Mullen PJ, Yu R, Longo J, Archer MC, Penn LZ (2016). The interplay between cell signalling and the mevalonate pathway in cancer. Nat. Rev. Cancer.

[7] Clendening JW (2010). Dysregulation of the mevalonate pathway promotes transformation. Proc. Natl Acad. Sci. USA..

[8] Boudreau DM (2004). A validation study of patient interview data and pharmacy records for antihypertensive, statin, and antidepressant medication use among older women. Am. J. Epidemiol..

[9] Moksud N (2021). Cholesterol-lowering drug use and breast cancer survival: the multiethnic cohort study. Breast Cancer Res. Treat..

[10] Ahern TP (2011). Statin prescriptions and breast cancer recurrence risk: a Danish nationwide prospective cohort study. J. Natl Cancer Inst..

[11] Cronin-Fenton D (2018). Breast cancer recurrence, bone metastases, and visceral metastases in women with stage II and III breast cancer in Denmark. Breast Cancer Res. Treat..

[12] Nielsen SF, Nordestgaard BG, Bojesen SE (2012). Statin use and reduced cancer-related mortality. N. Engl. J. Med..

[13] Kwan ML, Habel LA, Flick ED, Quesenberry CP, Caan B (2008). Post-diagnosis statin use and breast cancer recurrence in a prospective cohort study of early-stage breast cancer survivors. Breast Cancer Res. Treat..

[14] Brewer TM (2013). Statin use in primary inflammatory breast cancer: a cohort study. Br. J. Cancer.

[15] Lv H (2020). Association between statin use and prognosis of breast cancer: a meta-analysis of cohort studies. Front. Oncol..

[16] Chae YK (2011). Reduced risk of breast cancer recurrence in patients using ACE inhibitors, ARBs, and/or statins. Cancer Invest..

[17] Boudreau DM (2014). Comparative safety of cardiovascular medication use and breast cancer outcomes among women with early stage breast cancer. Breast Cancer Res. Treat..

[18] Goard CA (2014). Identifying molecular features that distinguish fluvastatin-sensitive breast tumor cells. Breast Cancer Res. Treat..

[19] Kimbung S, Lettiero B, Feldt M, Bosch A, Borgquist S (2016). High expression of cholesterol biosynthesis genes is associated with resistance to statin treatment and inferior survival in breast cancer. Oncotarget.

[20] Garwood ER (2010). Fluvastatin reduces proliferation and increases apoptosis in women with high-grade breast cancer. Breast Cancer Res. Treat..

[21] Bjarnadottir O (2013). Targeting HMG-CoA reductase with statins in a window-of-opportunity breast cancer trial. Breast Cancer Res. Treat..

[22] Clendening JW (2010). Exploiting the mevalonate pathway to distinguish statin-sensitive multiple myeloma. Blood.

[23] Longo J (2019). An actionable sterol-regulated feedback loop modulates statin sensitivity in prostate cancer. Mol. Metab..

[24] Brown MS, Goldstein JL (1997). The SREBP pathway: regulation of cholesterol metabolism by proteolysis of a membrane-bound transcription factor. Cell.

[25] Pandyra A (2014). Immediate utility of two approved agents to target both the metabolic mevalonate pathway and its restorative feedback loop. Cancer Res..

[26] Pandyra AA (2015). Genome-wide RNAi analysis reveals that simultaneous inhibition of specific mevalonate pathway genes potentiates tumor cell death. Oncotarget.

[27] Ye Y (2007). Enhanced cardioprotection against ischemia-reperfusion injury with dipyridamole and low-dose atorvastatin combination. Am. J. Physiol. Heart Circ. Physiol..

[28] Longo J (2020). Cyclic AMP-hydrolyzing phosphodiesterase inhibitors potentiate statin-induced cancer cell death. Mol. Oncol..

[29] El-Hachem N (2017). Integrative cancer pharmacogenomics to infer large-scale drug taxonomy. Cancer Res.

[30] Subramanian A (2017). A next generation connectivity map: l1000 platform and the first 1,000,000 profiles. Cell.

[31] Shoemaker RH (2006). The NCI60 human tumour cell line anticancer drug screen. Nat. Rev. Cancer.

[32] Wang B (2014). Similarity network fusion for aggregating data types on a genomic scale. Nat. Methods.

[33] Samal B (1978). Chromomycin A3 for advanced breast cancer: a Southwest Oncology Group study. Cancer Treat. Rep..

[34] Godt J (2006). The toxicity of cadmium and resulting hazards for human health. J. Occup. Med. Toxicol..

[35] Martirosyan A, Clendening JW, Goard CA, Penn LZ (2010). Lovastatin induces apoptosis of ovarian cancer cells and synergizes with doxorubicin: potential therapeutic relevance. BMC Cancer.

[36] Wu J, Wong WW-L, Khosravi F, Minden MD, Penn LZ (2004). Blocking the Raf/MEK/ERK pathway sensitizes acute myelogenous leukemia cells to lovastatin-induced apoptosis. Cancer Res.

[37] McGregor GH (2020). Targeting the metabolic response to statin-mediated oxidative stress produces a synergistic antitumor response. Cancer Res..

[38] Ianevski A, He L, Aittokallio T, Tang J (2017). SynergyFinder: a web application for analyzing drug combination dose-response matrix data. Bioinformatics.

[39] Haibe-Kains B (2012). A three-gene model to robustly identify breast cancer molecular subtypes. J. Natl Cancer Inst..

[40] Marcotte R (2016). Functional genomic landscape of human breast cancer drivers, vulnerabilities, and resistance. Cell.

[41] Liberzon A (2015). The Molecular Signatures Database (MSigDB) hallmark gene set collection. Cell Syst..

[42] Yu R (2018). Statin-induced cancer cell death can be mechanistically uncoupled from prenylation of ras family proteins. Cancer Res.

[43] Viswanathan VS (2017). Dependency of a therapy-resistant state of cancer cells on a lipid peroxidase pathway. Nature.

[44] Schormann, W., Hariharan, S. & Andrews, D. W. A reference library for assigning protein subcellular localizations by image-based machine learning. *J. Cell Biol*. **219**, (2020). 10.1083/jcb.201904090 PMC7055006 31968357

[45] Longo, J. et al. The mevalonate pathway is an actionable vulnerability of t(4;14)-positive multiple myeloma. *Leukemia*10.1038/s41375-020-0962-2 (2020). 10.1038/s41375-020-0962-2 PMC7359767 32665698

[46] Hay BA (2007). Aminopyrrolidineamide inhibitors of site-1 protease. Bioorg. Med. Chem. Lett..

[47] Longo J, van Leeuwen JE, Elbaz M, Branchard E, Penn LZ (2020). Statins as anticancer agents in the era of precision medicine. Clin. Cancer Res.

[48] Raghu VK (2018). Biomarker identification for statin sensitivity of cancer cell lines. Biochem. Biophys. Res. Commun..

[49] Held MA (2013). Genotype-selective combination therapies for melanoma identified by high-throughput drug screening. Cancer Disco..

[50] Gayvert KM (2017). A computational approach for identifying synergistic drug combinations. PLoS Comput. Biol..

[51] Levine BD, Cagan RL (2016). Drosophila lung cancer models identify trametinib plus statin as candidate therapeutic. Cell Rep..

[52] Beckwitt CH, Shiraha K, Wells A (2018). Lipophilic statins limit cancer cell growth and survival, via involvement of Akt signaling. PLoS ONE.

[53] Longo, J. et al. A pilot window-of-opportunity study of preoperative fluvastatin in localized prostate cancer. *Prostate Cancer Prostatic Dis*. 10.1038/s41391-020-0221-7 (2020). 10.1038/s41391-020-0221-7 PMC7655503 32203069

[54] Knuuttila E, Riikonen J, Syvälä H, Auriola S, Murtola TJ (2019). Access and concentrations of atorvastatin in the prostate in men with prostate cancer. Prostate.

[55] Bender AT, Beavo JA (2006). Cyclic nucleotide phosphodiesterases: molecular regulation to clinical use. Pharmacol. Rev..

[56] King AE, Ackley MA, Cass CE, Young JD, Baldwin SA (2006). Nucleoside transporters: from scavengers to novel therapeutic targets. Trends Pharmacol. Sci..

[57] Steinfelder HJ, Joost HG (1988). Inhibition of insulin-stimulated glucose transport in rat adipocytes by nucleoside transport inhibitors. FEBS Lett..

[58] Chow WA, Jiang C, Guan M (2009). Anti-HIV drugs for cancer therapeutics: back to the future?. Lancet Oncol..

[59] Guan M, Su L, Yuan Y-C, Li H, Chow WA (2015). Nelfinavir and nelfinavir analogs block site-2 protease cleavage to inhibit castration-resistant prostate cancer. Sci. Rep..

[60] Soprano M (2016). Oxidative stress mediates the antiproliferative effects of nelfinavir in breast cancer cells. PLoS One.

[61] Hitz F (2019). Nelfinavir and lenalidomide/dexamethasone in patients with lenalidomide-refractory multiple myeloma. A phase I/II Trial (SAKK 39/10). Blood Cancer J..

[62] Rengan R (2019). Clinical outcomes of the hiv protease inhibitor nelfinavir with concurrent chemoradiotherapy for unresectable stage iiia/iiib non–small cell lung cancer: a phase 1/2 trial. JAMA Oncol..

[63] Blumenthal GM (2014). A phase I trial of the HIV protease inhibitor nelfinavir in adults with solid tumors. Oncotarget.

[64] Hsyu P-H, Schultz-Smith MD, Lillibridge JH, Lewis RH, Kerr BM (2001). Pharmacokinetic interactions between nelfinavir and 3-hydroxy-3-methylglutaryl coenzyme a reductase inhibitors atorvastatin and simvastatin. Antimicrobial Agents Chemother..

[65] Beckwitt CH (2018). Statins attenuate outgrowth of breast cancer metastases. Br. J. Cancer.

[66] Marti JLG, Beckwitt CH, Clark AM, Wells A (2021). Atorvastatin facilitates chemotherapy effects in metastatic triple-negative breast cancer. Br. J. Cancer.

[67] Warita K (2014). Statin-induced mevalonate pathway inhibition attenuates the growth of mesenchymal-like cancer cells that lack functional E-cadherin mediated cell cohesion. Sci. Rep..

[68] Goss GD (2016). A phase I study of high-dose rosuvastatin with standard dose erlotinib in patients with advanced solid malignancies. J. Transl. Med..

[69] Hus M (2011). Thalidomide, dexamethasone and lovastatin with autologous stem cell transplantation as a salvage immunomodulatory therapy in patients with relapsed and refractory multiple myeloma. Ann. Hematol..

[70] Knox JJ (2005). A Phase I trial of prolonged administration of lovastatin in patients with recurrent or metastatic squamous cell carcinoma of the head and neck or of the cervix. Eur. J. Cancer.

[71] Kornblau SM (2007). Blockade of adaptive defensive changes in cholesterol uptake and synthesis in AML by the addition of pravastatin to idarubicin + high-dose Ara-C: a phase 1 study. Blood.

[72] Murtola TJ (2018). Atorvastatin versus placebo for prostate cancer before radical prostatectomy—a randomized, double-blind, placebo-controlled clinical trial. Eur. Urol..

[73] Tanimoto, T. T. *An Elementary Mathematical Theory of Classification and Prediction*. (International Business Machines Corporation, 1958).

[74] Guha R (2007). & Others. Chemical informatics functionality in R. J. Stat. Softw..

[75] Smirnov P (2016). PharmacoGx: an R package for analysis of large pharmacogenomic datasets. Bioinformatics.

[76] Subramanian T (2005). & Others. Molecular Signatures Database (MSigDB). Proc. Natl Acad. Sci. USA..

[77] Romero P (2005). Computational prediction of human metabolic pathways from the complete human genome. Genome Biol..

[78] Kanehisa M, Goto S (2000). KEGG: kyoto encyclopedia of genes and genomes. Nucleic Acids Res..

[79] Du J (2014). KEGG-PATH: Kyoto encyclopedia of genes and genomes-based pathway analysis using a path analysis model. Mol. Biosyst..

[80] Caspi R (2016). The MetaCyc database of metabolic pathways and enzymes and the BioCyc collection of pathway/genome databases. Nucleic Acids Res..

[81] Csardi G, Nepusz T (2006). & Others. The igraph software package for complex network research. InterJournal, complex Syst..

[82] Gehlenborg, N. UpSetR: a more scalable alternative to Venn and Euler diagrams for visualizing intersecting sets. *R package version* **1**, (2019).

[83] Wickham, H. pryr: Tools for Computing on the Language. (2018).

[84] Graham FL, Smiley J, Russell WC, Nairn R (1977). Characteristics of a human cell line transformed by DNA from human adenovirus type 5. J. Gen. Virol..

[85] Bray NL, Pimentel H, Melsted P, Pachter L (2016). Near-optimal probabilistic RNA-seq quantification. Nat. Biotechnol..

[86] Frankish A (2019). GENCODE reference annotation for the human and mouse genomes. Nucleic Acids Res..

[87] Wirapati P (2008). Meta-analysis of gene expression profiles in breast cancer: toward a unified understanding of breast cancer subtyping and prognosis signatures. Breast Cancer Res..

[88] Gendoo DMA (2016). Genefu: an R/Bioconductor package for computation of gene expression-based signatures in breast cancer. Bioinformatics.

[89] Vichai V, Kirtikara K (2006). Sulforhodamine B colorimetric assay for cytotoxicity screening. Nat. Protoc..

[90] Subramanian A (2005). Gene set enrichment analysis: a knowledge-based approach for interpreting genome-wide expression profiles. Proc. Natl Acad. Sci. USA..

[91] Liberzon A (2011). Molecular signatures database (MSigDB) 3.0. Bioinformatics.

[92] Väremo L, Nielsen J, Nookaew I (2013). Enriching the gene set analysis of genome-wide data by incorporating directionality of gene expression and combining statistical hypotheses and methods. Nucleic Acids Res.

[93] Ashburner M (2000). Gene ontology: tool for the unification of biology. The gene ontology consortium. Nat. Genet..

[94] Sarrió D (2008). Epithelial-mesenchymal transition in breast cancer relates to the basal-like phenotype. Cancer Res.

[95] McQuin C (2018). CellProfiler 3.0: next-generation image processing for biology. PLoS Biol..

[96] Sachs N (2018). A living biobank of breast cancer organoids captures disease heterogeneity. Cell.

