# Supplementary material for: Computational pharmacogenomic screen identifies drugs that potentiate the anti-breast cancer activity of statins
Source: Nat Commun. 2022 Oct 24;13:6323. doi: 10.1038/s41467-022-33144-9 (PMC9592602; doi:10.1038/s41467-022-33144-9)
Supplement: Supplementary file 1 — Supplementary Information [file 41467_2022_33144_MOESM1_ESM.pdf]

SUPP FIGURE 1

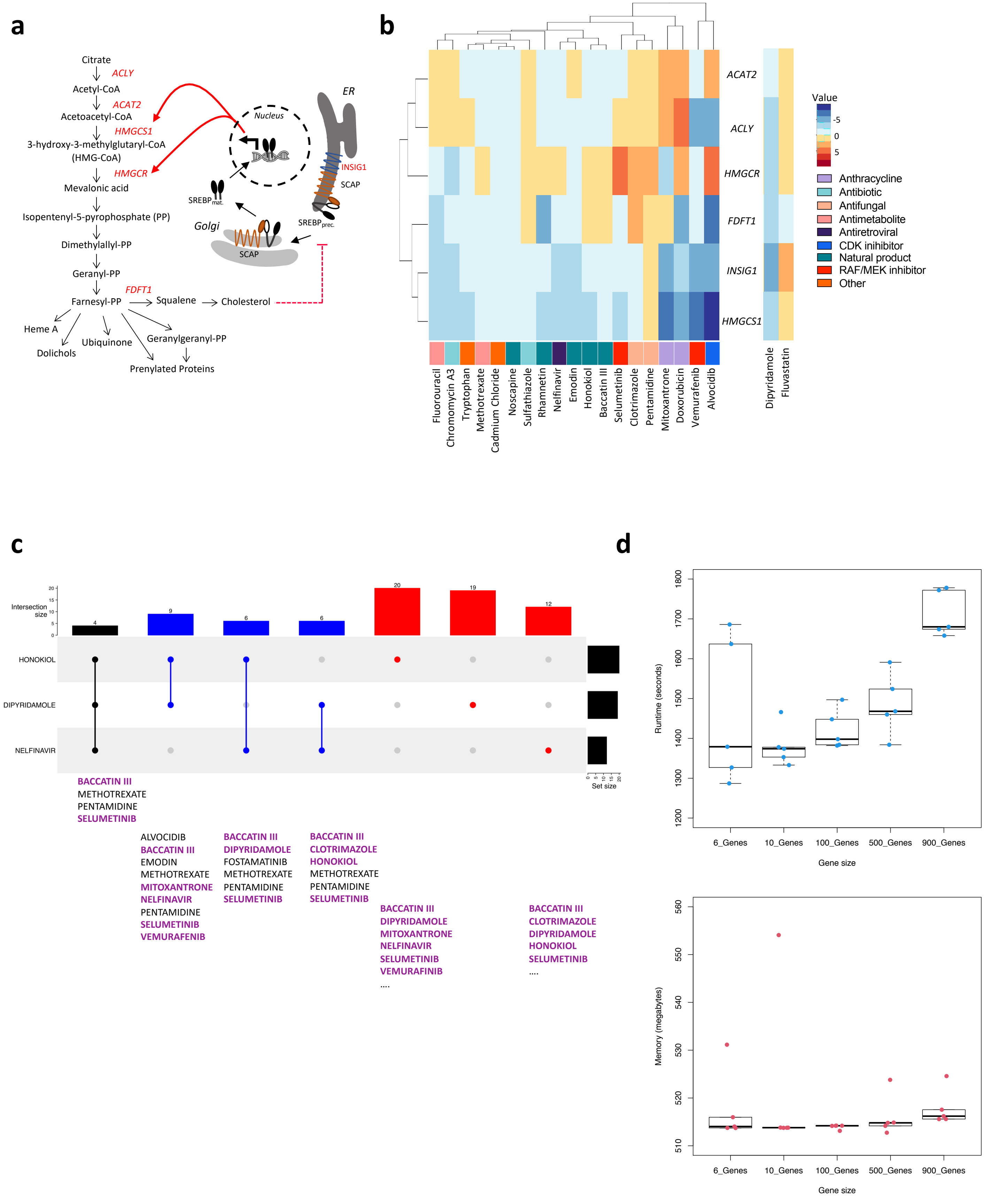

Figure. S1, related to Figure. 1. Additional information regarding drug-induced genotype changes and categorization of top 19 compounds. (A) Simplified schematic of the MVA pathway, highlighting the six MVA pathway genes (in red) in the L1000 database used to restrict the drug-induced gene perturbation layer of the MVA-DNF method. **(B)** Drug perturbation signatures for dipyridamole and identified compounds, plotted for genes pertaining to the MVA pathway. Similarity between compounds based on their overall expression profiles is rendered in the dendrogram. Dipyridamole- and fluvastatin-induced changes are shown on the side as reference. Categorization of the top 19 compounds is shown along the bottom, excluding toxins and carcinogenic compounds. **(C)** Upset plot indicating the overlap of top drug hits for the MVA-DNF when using each of dipyridamole, nelfinavir, or honokiol as the prototype drug for testing. Top drug hits to each of the drugs were selected based on significance ($p \leq 0.05$ and z-score <-1.8); computation of the z-score and p-value are described in the methods Nelfinavir and dipyridamole are identified as top hits to honokiol, and honokiol and dipyridamole were identified as top drug hits to nelfinavir (red bars). Comparison of the overlaps between drug hits across the three drugs (black and blue bars) indicates that MVA-DNF converges on the same set of drugs, demonstrating selectivity for the MVA pathway. **(D)** Time consumption (CPU runtime in seconds) and memory consumption (in megabytes) of the MVA-DNF and permutation testing pipeline. N=5 complete and independent runs of the pipeline were performed across different gene sizes using a random number of 6, 10, 100, 500 and 900 genes in the drug perturbation layer. Each individual run is composed of the MVA-DNF and 999 random iterations of the MVA-DNF network with the designated gene size in the drug perturbation layer. The minimum, maximum, and interquartile range of the CPU runtime and memory consumption across these runs are delineated using the box-and-whiskers plot; individual experiments are indicated as blue points (for the CPU runtime) and red points (for the memory consumption).

SUPP FIGURE 2

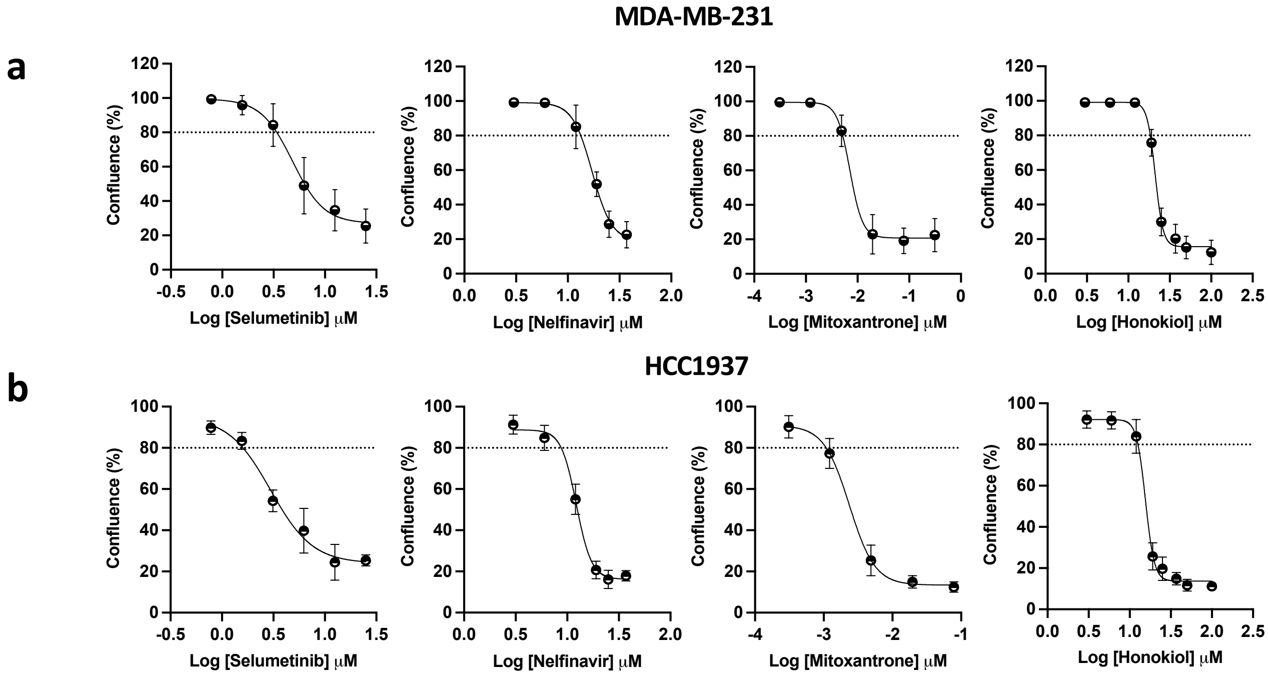

Figure. S2. Drug-dose response curves for MDA-MB-231 and HCC1937 breast cancer cell lines to identify a sub-lethal dose of top MVA-DNF compounds. Dose-response curves of selumetinib, nelfinavir, mitoxantrone and honokiol in **(A)** MDA-MB-231 and **(B)** HCC1937 cell lines are determined using the IncuCyte Live-Cell Imaging System. Cell lines were cultured with increasing concentrations of selumetinib, nelfinavir, mitoxantrone or honokiol for 5 days. Cell confluence was calculated using IncuCyte Zoom instrument software from the phase contrast channel. The mean and standard deviation (SD) for each drug concentration from 3 independent biological replicates is plotted. Doses that impede proliferation, but do not have a strong single-agent effect (i.e. <20% effect on cell confluence) were carried forward for further assays.

SUPP FIGURE 3 (HCC1937)

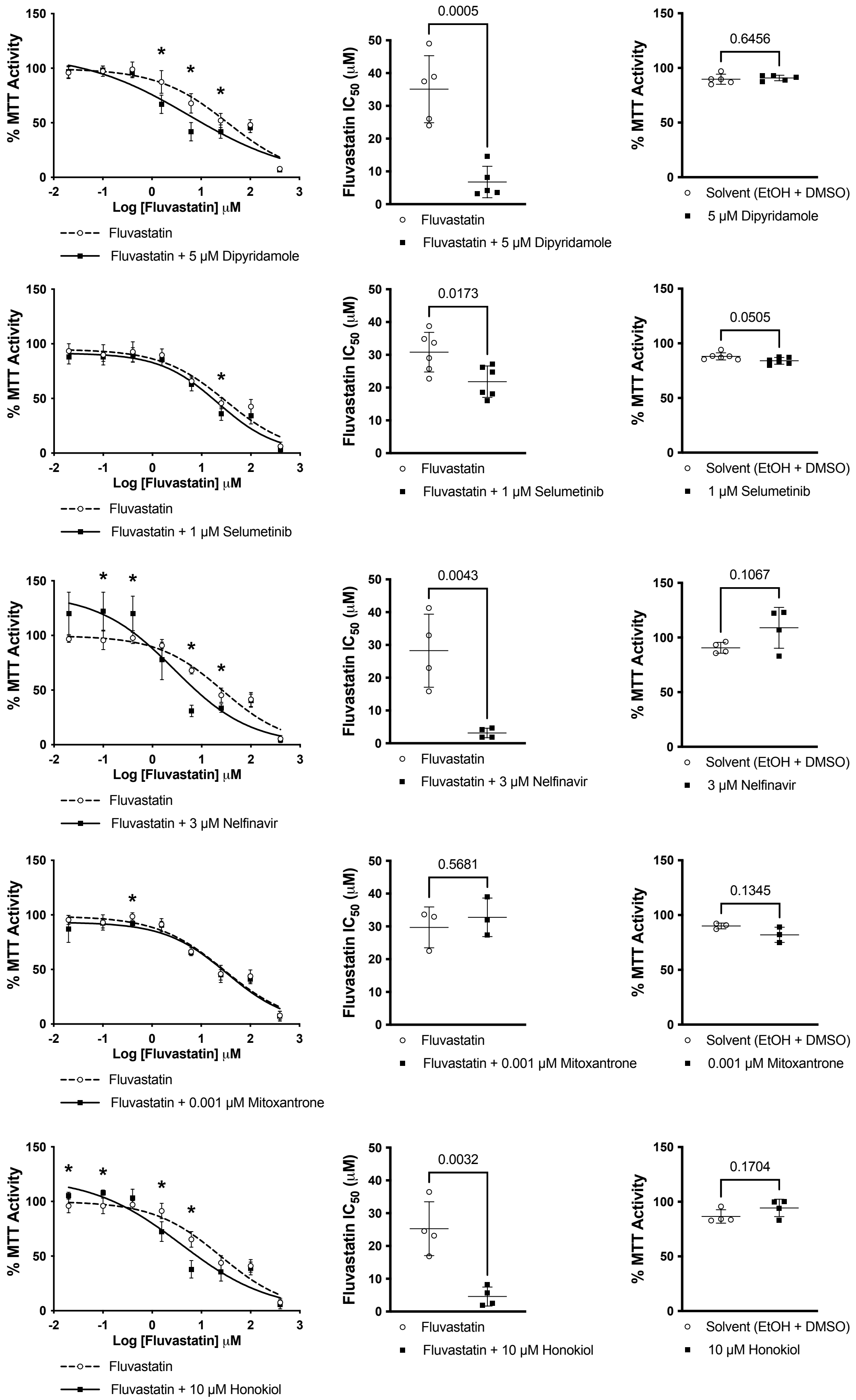

Figure. S3. Drug-dose response curves, fluvastatin IC_{50} and solvent control values for HCC1937 cells. HCC1937 cells were treated with a range of fluvastatin doses alone or in combination with a sub-lethal dose of dipyridamole (5 µM), selumetinib (1 µM), nelfinavir (3 µM), mitoxantrone (0.001 µM) or honokiol (10 µM) for 72 hours, and cell viability was determined using an MTT assay. The drug dose-response curves (left), fluvastatin IC_{50} values (middle) and control values (right) are plotted with p-values for the latter two graphs shown above the bracket. Error bars represent the mean +/- SD, n = 3-6 biologically independent experiments, *p <0.05 (Student *t* test, unpaired, two-tailed).

SUPP FIGURE 4 (MDA-MB-231)

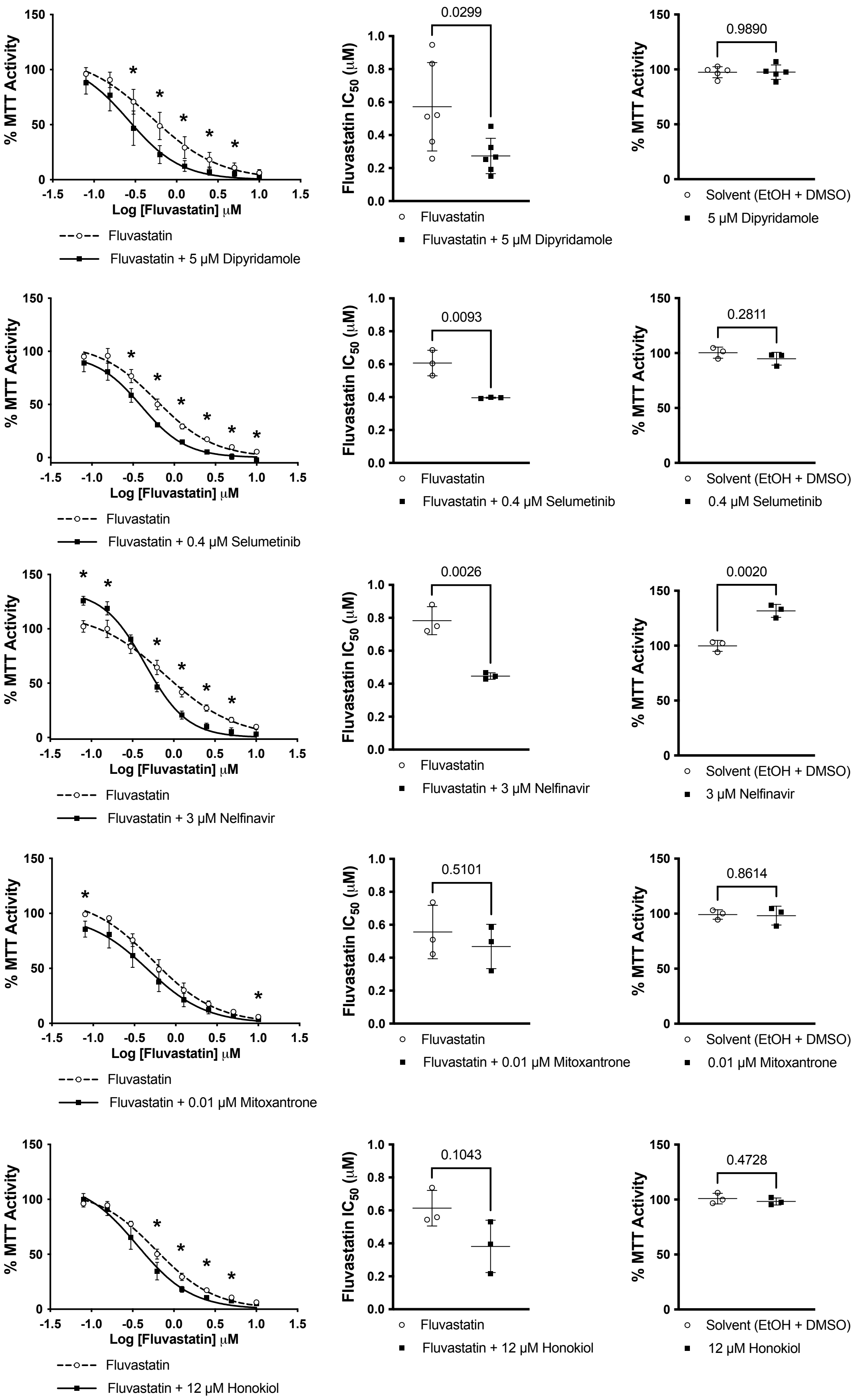

Figure. S4. Drug-dose response curves, fluvastatin IC_{50} and solvent control values for MDA-MB-231 cells. MDA-MB-231 cells were treated with a range of fluvastatin doses alone or in combination with a sub-lethal dose of dipyridamole (5 μM), selumetinib (0.4 μM), nelfinavir (3 μM), mitoxantrone (0.01 μM) or honokiol (12 μM) for 72 hours, and cell viability was determined using an MTT assay. The drug dose-response curves (left), fluvastatin IC_{50} values (middle) and control values (right) are plotted with p-values for the latter two graphs shown above the bracket. Error bars represent the mean +/- SD, n = 3-5 biologically independent experiments, *p <0.05 (Student *t* test, unpaired, two-tailed).

SUPP FIGURE 5

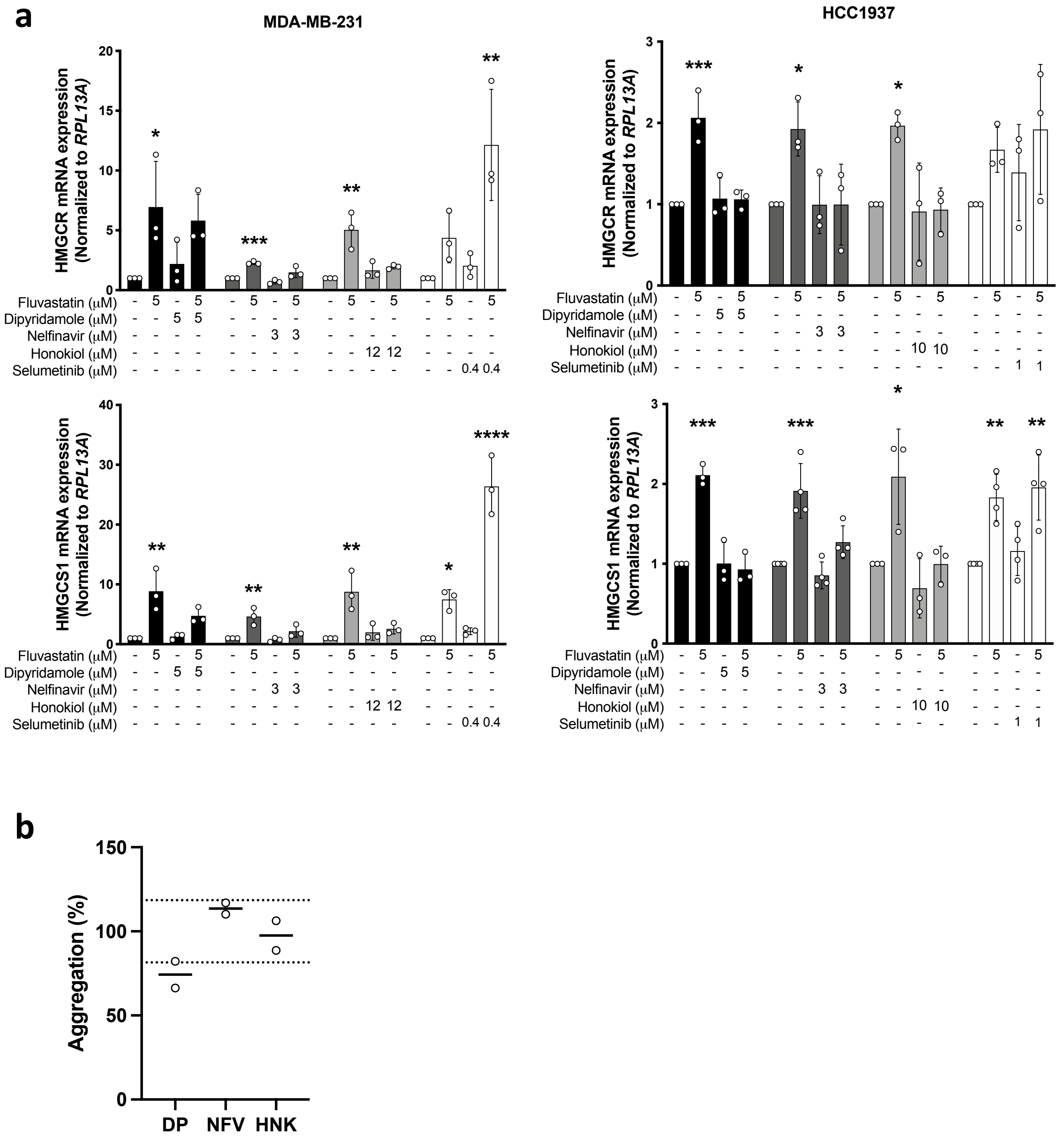

Figure. S5, related to Figure 3. Nelfinavir and Honokiol block fluvastatin-induced activation of SREBP2 target genes. (A) MDA-MB-231 (left) and HCC1937 (right) cells were treated with fluvastatin +/- dipyridamole, nelfinavir, honokiol or selumetinib for 16 hours, and RNA was isolated to assay for *HMGCR* and *HMGCS1* expression by qRT-PCR. mRNA expression data are normalized to *RPL13A* expression. Error bars represent the mean +/- SD, n = 3 biologically independent samples, *p <0.05, **p <0.01, ***p <0.001, ****p <0.0001 (one-way ANOVA with Bonferroni's multiple comparisons test, where each group was compared to the solvent control group within each experiment). **(B)** Dipyridamole (10 μM) inhibits thrombin receptor activating peptide (TRAP-6)-induced platelet aggregation in whole blood. Nelfinavir (10 μM) and honokiol (10 μM) did not block TRAP-6-induced aggregation. Samples were incubated with each compound for 3 minutes then treated platelets were activated with 32 μM TRAP-6. Aggregation over 10 minutes was measured using a Multiplate Analyzer and expressed as a percentage of aggregation by TRAP-6 alone in that donor. Error bars represent the average +/- SD, n = 2. Dashed lines represent SD of aggregation with TRAP-6 alone. *p <0.05, **p<0.01, ***p<0.001, ****p<0.0001 (one-way ANOVA with Bonferroni's multiple comparisons test, where each group was compared to the solvent controls group).

SUPP FIG 6

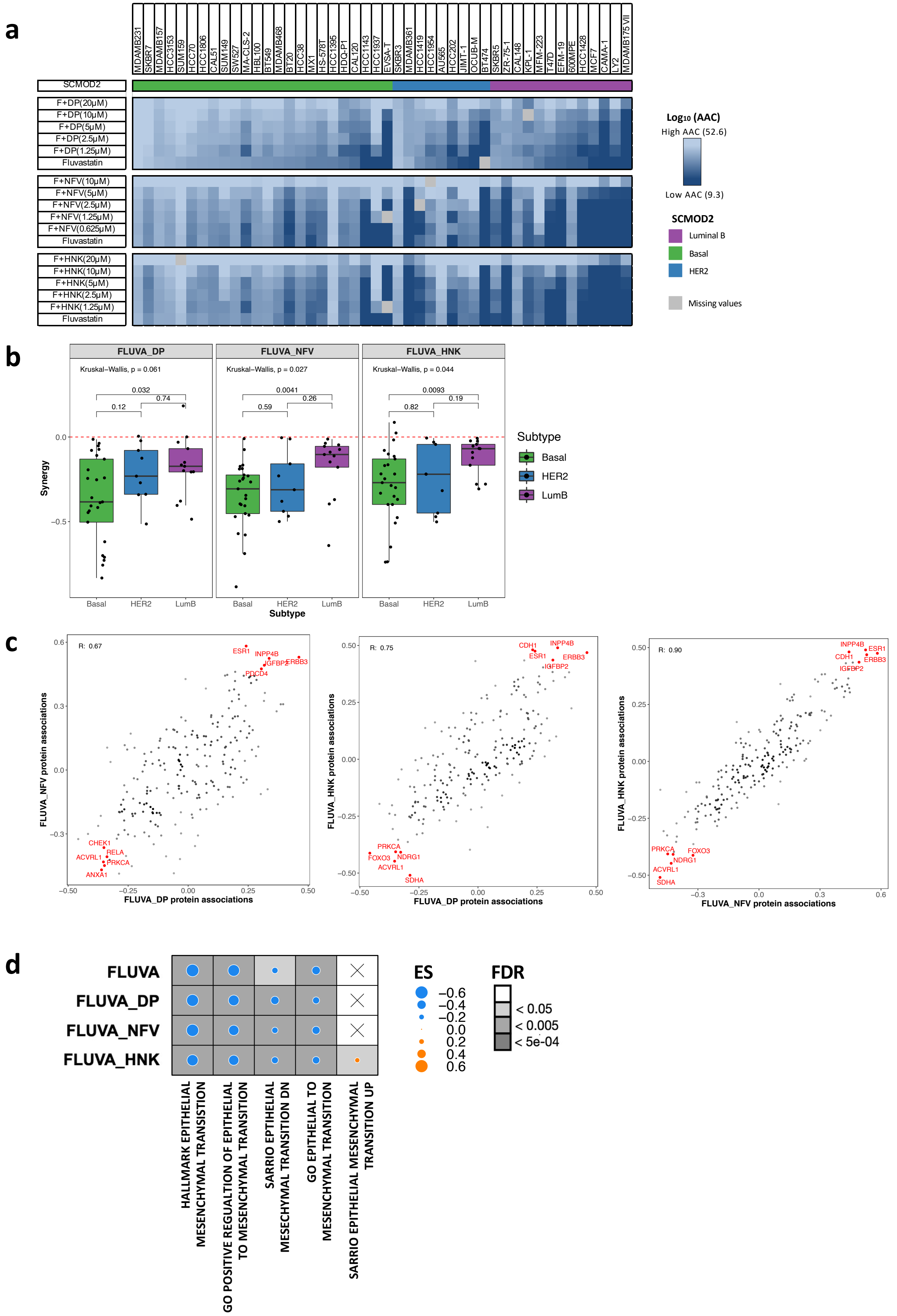
a
MDAMB231
SKBR7
MDAMB157
HCC3153
SUM159
HCC70
HCC1806
CAL51
SUM149
SW527
MA-CLS-2
HBL100
BT549
MDAMB468
BT20
HCC38
MX1
HS-578T
HCC1395
HDQ-P1
CAL120
HCC1143
HCC1937
EVSA-T
SKBR3
MDAMB361
HCC1419
HCC1954
AU565
HCC202
JIMT-1
OCUB-M
BT474
SKBR5
ZR-75-1
CAL148
KPL-1
MFM-223
T47D
EFM-19
600MPE
HCC1428
MCF7
CAMA-1
LY2
MDAMB175VII
SCMOD2
F+DP(20μM)
F+DP(10μM)
F+DP(5μM)
F+DP(2.5μM)
F+DP(1.25μM)
Fluvastatin
F+NFV(10μM)
F+NFV(5μM)
F+NFV(2.5μM)
F+NFV(1.25μM)
F+NFV(0.625μM)
Fluvastatin
F+HNK(20μM)
F+HNK(10μM)
F+HNK(5μM)
F+HNK(2.5μM)
F+HNK(1.25μM)
Fluvastatin
Log10 (AAC)
High AAC (52.6)
Low AAC (9.3)
SCMOD2
Luminal B
Basal
HER2
Missing values
b
FLUVA_DP
FLUVA_NFV
FLUVA_HNK
Kruskal–Wallis, p = 0.061
Kruskal–Wallis, p = 0.027
Kruskal–Wallis, p = 0.044
0.032
0.74
0.12
0.0041
0.26
0.59
0.0093
0.19
0.82
Synergy
0.0
–0.5
Basal
HER2
LumB
Subtype
Basal
HER2
LumB
c
R: 0.67
R: 0.75
R: 0.90
ESR1
INPP4B
IGFBP2
ERBB3
PDCD4
CHEK1
RELA
ACVRL1
PRKCA
ANXA1
CDH1
FOXO3
NDRG1
SDHA
FLUVA_NFV protein associations
FLUVA_HNK protein associations
FLUVA_DP protein associations
d
FLUVA
FLUVA_DP
FLUVA_NFV
FLUVA_HNK
HALLMARK EPITHELIAL MESENCHYMAL TRANSISTION
GO POSITIVE REGUALTION OF EPITHELIAL TO MESENCHYMAL TRANSITION
SARRIO EPTIHELIAL MESECHYMAL TRANSITION DN
GO EPITHELIAL TO MESENCHYMAL TRANSITION
SARRIO EPITHELIAL MESENCHYMAL TRANSITION UP
ES
–0.6
–0.4
–0.2
0.0
0.2
0.4
0.6
FDR
< 0.05
< 0.005
< 5e-04

Figure S6, related to Figure 4. High-throughput compound combination screen. (A) Heatmap of Log_{10}(AAC) values for a high-throughput compound synergy screen against 47 BC cell lines visualizing the 15th to 85th percentile. BC cell lines were treated with a dose matrix of fluvastatin (F) (0-20 μM) +/- dipyridamole (DP) (0-20 μM), nelfinavir (NFV) (0-10 μM) or honokiol (HNK) (0-20 μM). After 5 days of drug treatment, cell viability was assessed by SRB assay. SCMOD2 cell line subtyping was assigned to the BC cell line panel. Data presented are the average of 2 biological replicates (F +/- DP) or the mean of 3-6 biological replicates (F +/- NFV and F +/- HNK) and ordered by average fluvastatin AAC and SCMOD2 subtype. **(B)** Comparison of Bliss synergy scores stratified by BC subtypes across the combinations using two-sided wilcoxon paired rank test. Red dashed line at synergy threshold where >0 indicates lower synergy and <0 indicates higher synergy. p-values shown above the bracket (Kruskal-Wallis test). The center lines, bounds of box, whiskers, points of boxplot indicate median, lower/upper quartile (25th/75th percentile), minima/maxima, and raw data, respectively. **(C)** Associations of proteomic states[40] with synergy scores across the fluvastatin-compound combinations. Similarity of proteomic states associations were compared across the combinations (Fluva-DP vs Fluva-NFV; Fluva-DP vs Fluva-HNK; Fluva-NFV vs Fluva-HNK) using Pearson correlation coefficient. Top five basally-expressed proteins associated with synergy in either direction are annotated in red. **(D)** Gene set enrichment analysis using five EMT gene set collections and genes ranked by basal mRNA correlated to the fluvastatin IC_{50} (Fluva) value or synergy score (Fluva-DP, Fluva-NFV and Fluva-HNK). Dot size indicates the difference in enrichment scores (ES) of the pathways. Background shading indicates the FDR. X indicates pathway and drug combinations that were not significantly enriched (FDR > 0.05).

SUPP FIG 7

a

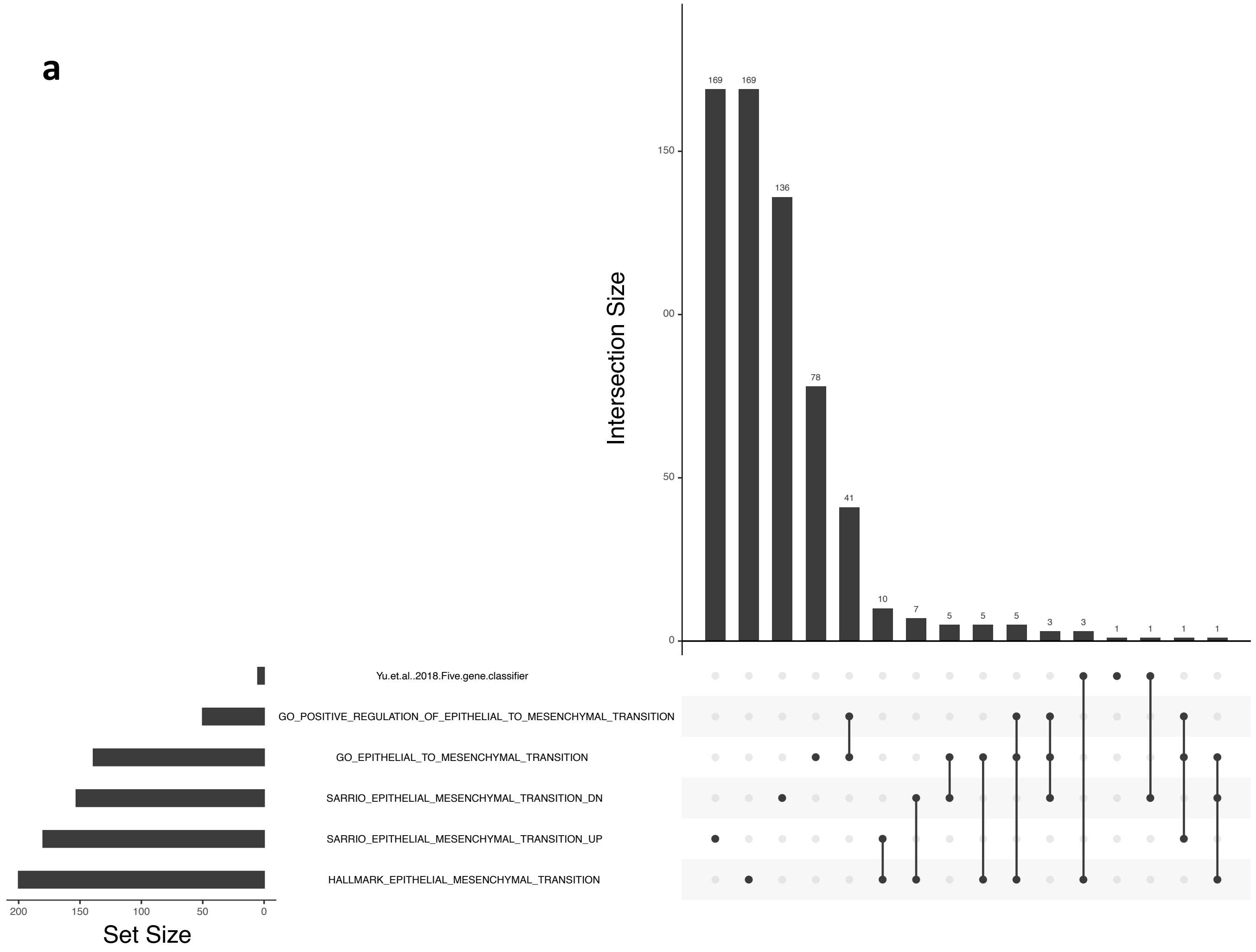

b

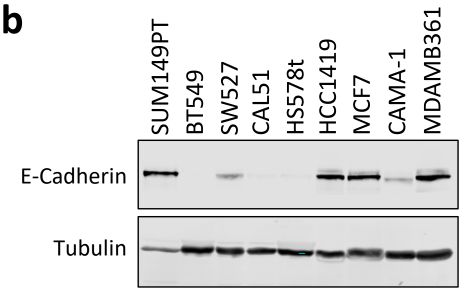

Figure S7, related to Figure 4. Overlapping genes within the EMT gene sets. (A) Upset plot to visualize the agreement between Yu *et al.* (2018)[42] five-gene classifier and five additional EMT gene sets. **(B)** Protein lysates were isolated from a panel of BC cell lines to assay for basal E-cadherin expression by immunoblotting. N=3 biologically independent experiments, representative blot is shown. Source data are provided as source data file.

SUPP FIG 8

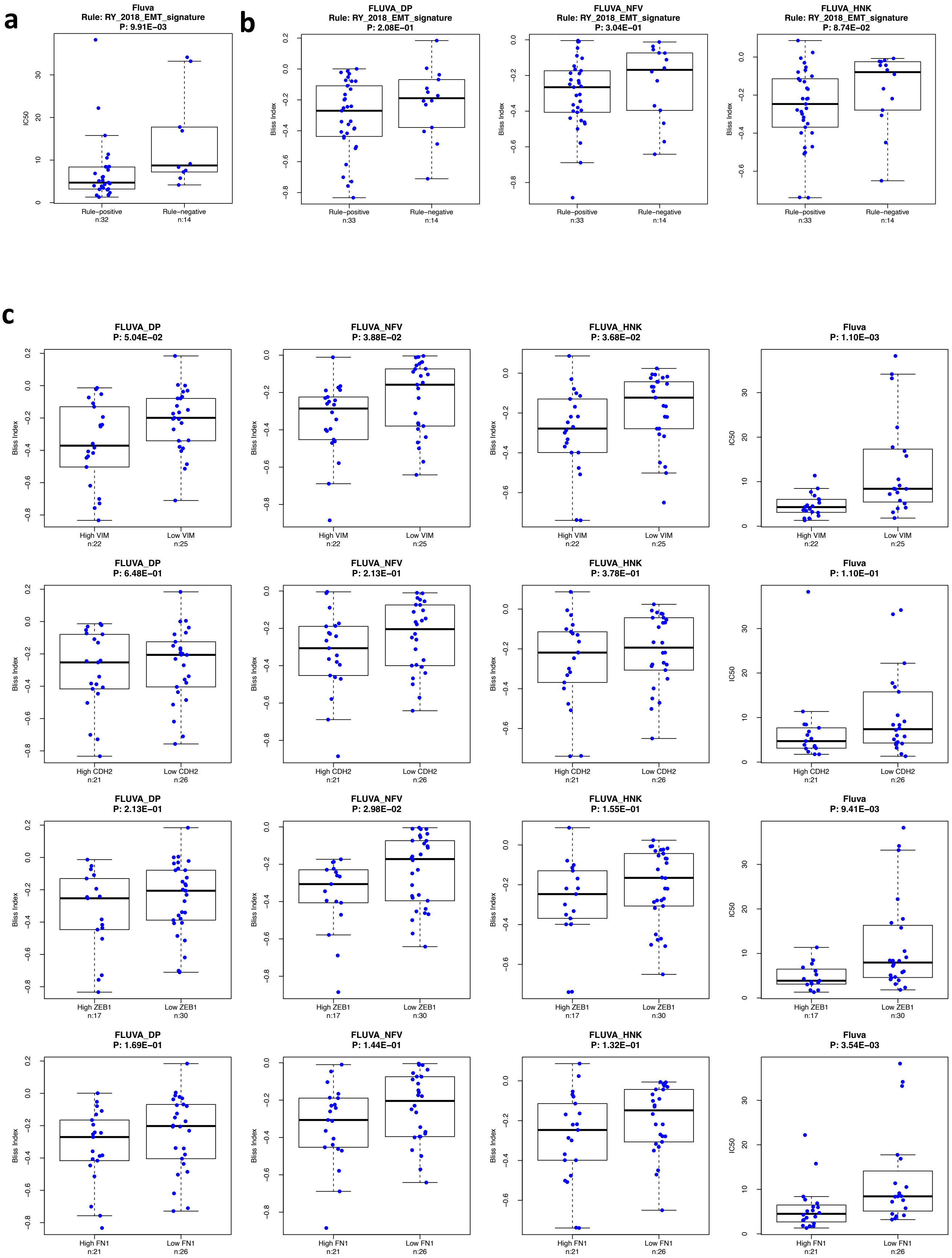

a
Fluva
Rule: RY_2018_EMT_signature
P: 9.91E−03
IC50
Rule−positive n:32
Rule−negative n:14
b
FLUVA_DP
Rule: RY_2018_EMT_signature
P: 2.08E−01
FLUVA_NFV
Rule: RY_2018_EMT_signature
P: 3.04E−01
FLUVA_HNK
Rule: RY_2018_EMT_signature
P: 8.74E−02
Bliss Index
Rule−positive n:33
Rule−negative n:14
c
FLUVA_DP P: 5.04E−02
FLUVA_NFV P: 3.88E−02
FLUVA_HNK P: 3.68E−02
Fluva P: 1.10E−03
High VIM n:22
Low VIM n:25
FLUVA_DP P: 6.48E−01
FLUVA_NFV P: 2.13E−01
FLUVA_HNK P: 3.78E−01
Fluva P: 1.10E−01
High CDH2 n:21
Low CDH2 n:26
FLUVA_DP P: 2.13E−01
FLUVA_NFV P: 2.98E−02
FLUVA_HNK P: 1.55E−01
Fluva P: 9.41E−03
High ZEB1 n:17
Low ZEB1 n:30
FLUVA_DP P: 1.69E−01
FLUVA_NFV P: 1.44E−01
FLUVA_HNK P: 1.32E−01
Fluva P: 3.54E−03
High FN1 n:21
Low FN1 n:26
Bliss Index
IC50

Figure S8, related to Figure 4. EMT gene expression as a biomarker of sensitivity to fluvastatin and synergistic response to fluvastatin-compound combinations. **(A)** Five-gene fluvastatin sensitivity gene classifier[42] predicts sensitivity to fluvastatin alone, but **(B)** does not predict synergy to Fluva-DP, Fluva-NFV or Fluva-HNK. **(C)** Basal Vimentin (VIM), N-Cadherin (CDH2), ZEB1 and fibronectin (FN1) mRNA expression do not predict synergy to the drug combinations. Gene expression was binarized based on Z-score. P-value was measured by two-sided wilcoxon rank sum test. The center lines, bounds of box, whiskers, points of boxplot indicate median, lower/upper quartile (25th/75th percentile), minima/maxima, and raw data, respectively.

SUPP FIGURE 9

a

BPTO.66

Bliss synergy score: 8.029

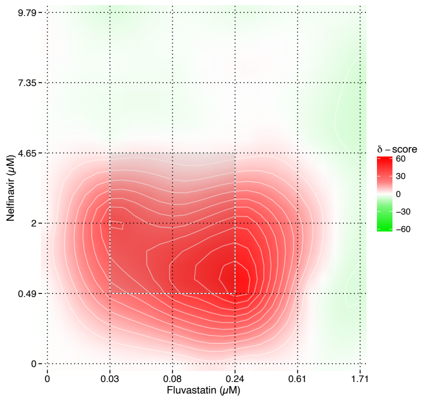

b

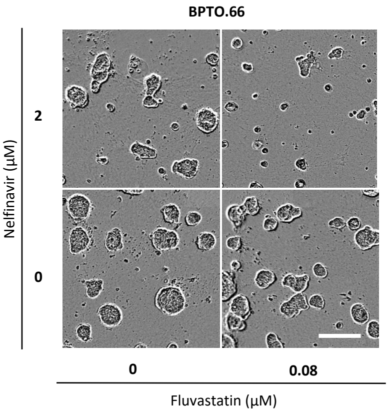

BPTO.95

Bliss synergy score: 5.978

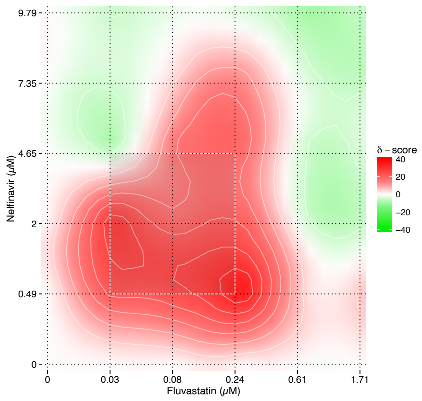

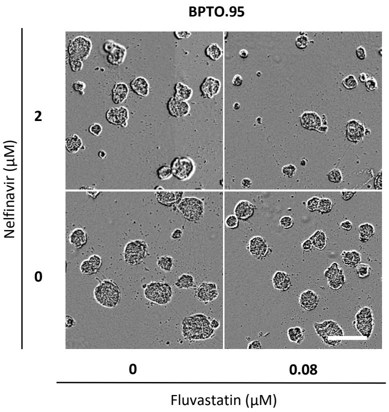

BPTO.51

Bliss synergy score: 16.479

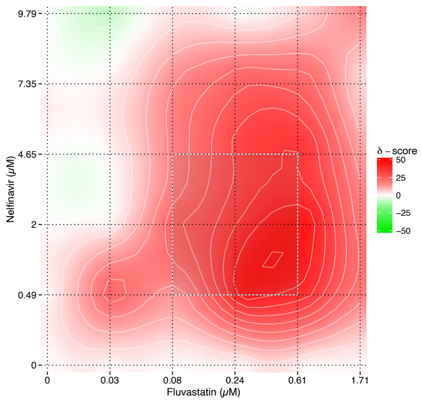

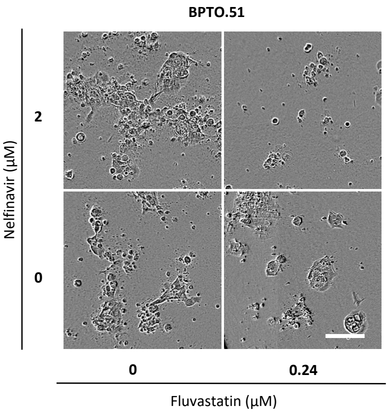

Figure S9, related to Figure 5. Bliss synergy scores for patient derived breast organoids. (A) Synergy plots for BPTO.66, BPTO.95 and BPTO.51 organoids treated with the indicated doses of fluvastatin and nelfinavir, where red represents synergy and green represents antagonism. **(B)** Representative images of BPTO.66, BPTO.95 and BPTO.51 organoids are shown after 14 days of treatment. N = 2-5 biologically independent experiments. Selected images represent the most synergistic area identified through SynergyFinder. Scale bars = 200 μm.

SUPP FIGURE 10

a

Compound doses (μM)

	DMSO	Dipyridamole	Nelfinavir	Honokiol	Vemurafenib	Clotrimazole	Baccatin III	Pentamidine isethionate	Noscapine	Methotrexate	Fluorouracil	Alvocidib
Low		1	1	5	0.005	1	0.005	1	0.1	0.001	0.1	0.005
Medium		5	3	10	0.01	2	0.01	2	1	0.01	1.5	0.01
High		10	6	20	0.02	3	0.02	10	5	0.05	3	0.02

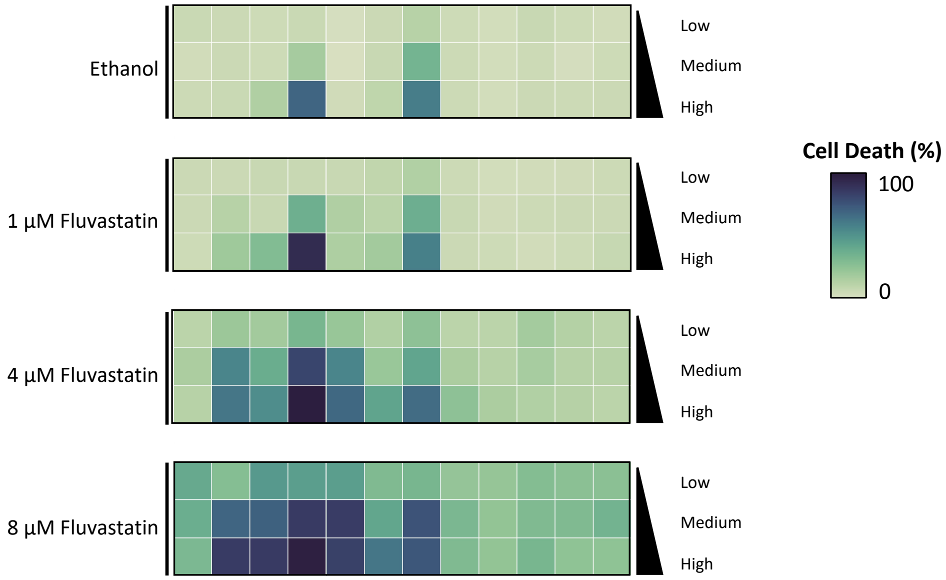

b

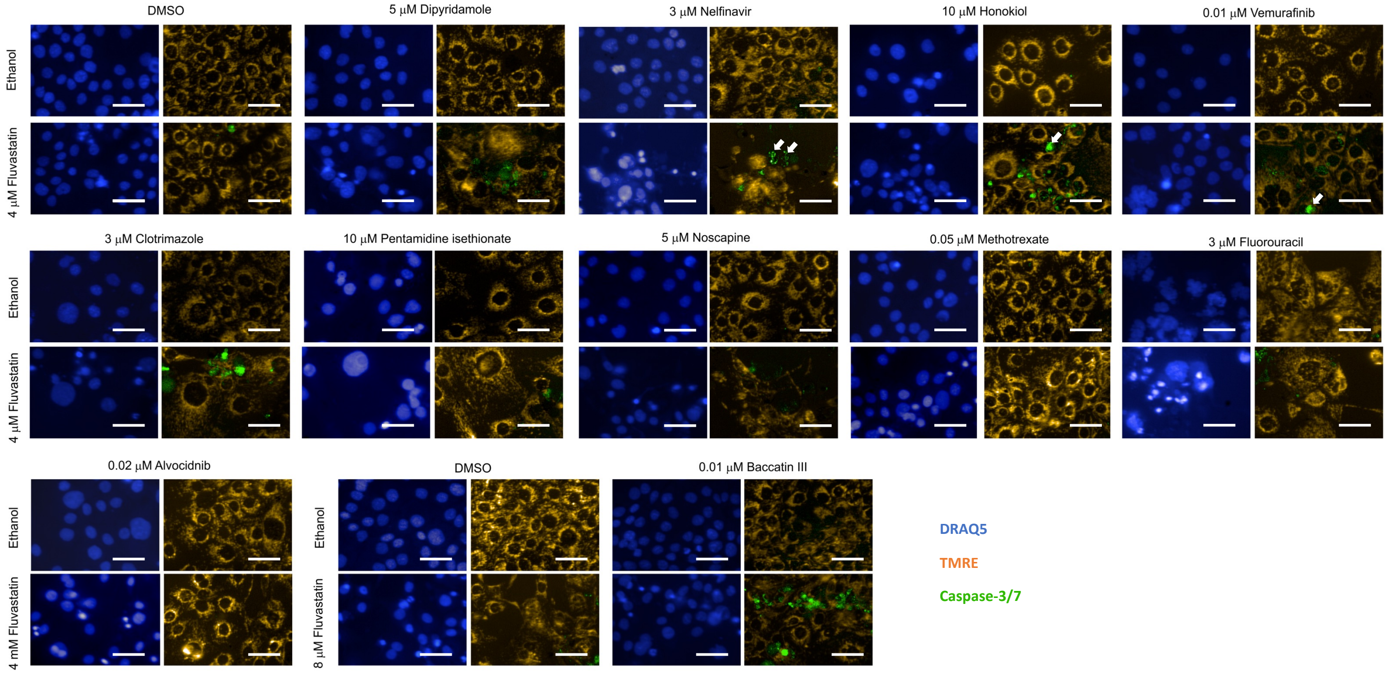

Figure S10, related to Figure 6. Live-cell imaging death assay. (A) HCC1937 cells were treated with solvent control (DMSO), 1 µM, 4 µM or 8 µM fluvastatin, MVA-DNF compound of interest or the combination for 72 hours. **(B)** Cells were then stained with DRAQ5 (blue, DNA dye), TMRE (orange, marker of active mitochondria) and Caspase-3/7 (green, marker of apoptotic cells). Stained cells were imaged by confocal microscopy. White arrows indicate cells that show TMRE loss. N = 4 biologically independent experiments. Scale bar = 50 µm.

SUPP FIGURE 11

U18666A

a

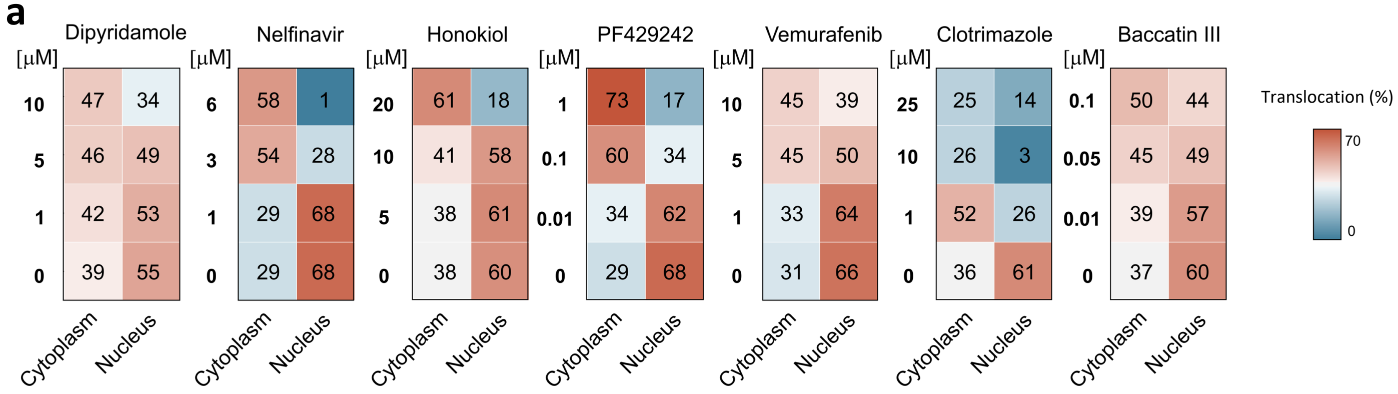

b

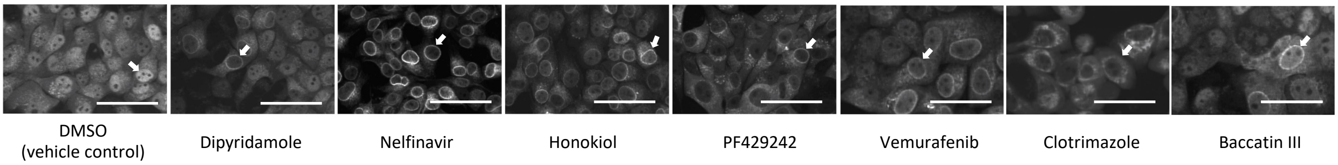

Figure S11, related to Figure 6. MVA-DNF compounds block SREBP2 translocation induced by fluvastatin and GGPP in lipoprotein deficient serum. (A) Classification result of subcellular localization of mNeon-SREBP2 in NMuMG cells in presence of U18666A (10 μM) treated with a set of compounds (high, medium, low dose) for 16 hrs. Treatment was carried out in 5% lipoprotein-deficient serum (LPDS)-supplemented culture media. Numbers shown within the heat map indicate the percentage of cells assigned either to cytoplasm or nucleus. While RanGTP functions as a nuclear landmark, Cytoplasm comprises multiple organelle markers: endoplasmic reticulum, Golgi apparatus, nuclear envelope, and secretory pathway. Results shown are representative of three replicates. **(B)** Sample micrographs of mNeon-SREBP2 expressed in NMuMG cells treated with U18666A in LPDS and the respective highest dose of dipyridamole, nelfinavir, honokiol, PF429242, vemurafenib, clotrimazole or baccatin III for 16 hrs. White arrow indicates nuclei of interest. N = 3 biologically independent experiments. Scale bar = 50 μm.

Supplementary Table 1. Ranked MVA-DNF compounds. Drug structure, z-score and p-values are indicated. Compounds are ordered by p-value and restricted to Z-score <-1.8.

Hits	Drug structure	Permutation Test		Currently used in humans	Currently used in cancer treatment	In clincal trials for cancer treatment	Pre-clinical	Research tool	Exclude	Classification
		Z-Score	P-Value*							
SELUMETINIB	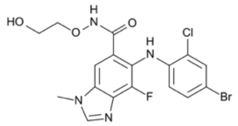	-3.57	1.77E-04	+		+				RAF/MEK inhibitor
NELFINAVIR	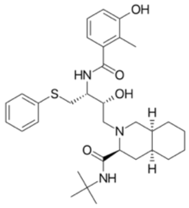	-3.28	5.18E-04	+		+				Antiretroviral
MITOXANTRONE	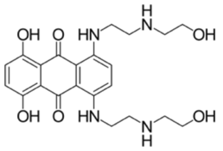	-3.20	6.82E-04	+	+	+				Anthracycline
DOXORUBICIN	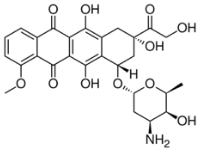	-3.03	1.22E-03	+	+	+				Anthracycline
HONOKIOL	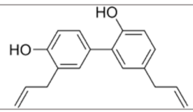	-2.96	1.53E-03				+			Natural product
CLOTRIMAZOLE	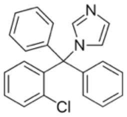	-2.95	1.57E-03	+						Antifungal
SULFATHIAZOLE	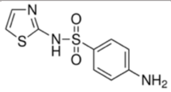	-2.89	1.90E-03				+			Antibiotic
VEMURAFENIB	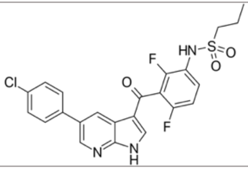	-2.67	3.79E-03	+	+	+				RAF/MEK inhibitor
CHROMOMYCINA3	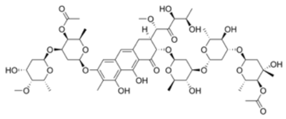	-2.64	4.13E-03				+	+	Toxin	Antibiotic
BACCATINIII	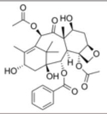	-2.53	5.69E-03				+			Natural product

**NOSCAPINE**	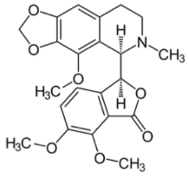	-2.51	6.11E-03			+	+			Natural product
**METHOTREXATE**	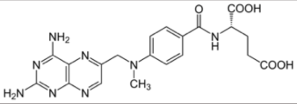	-2.29	1.11E-02	+	+	+				Antimetabolite
**CADMIUMCHLORIDE**		-2.22	1.33E-02				+	+	Carcinogen	Other
**RHAMNETIN**	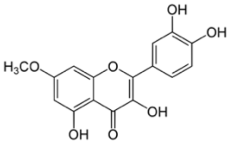	-2.18	1.45E-02				+			Natural product
**PENTAMIDINE**	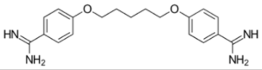	-2.18	1.46E-02	+		+				Antifungal
**EMODIN**	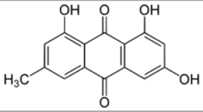	-2.16	1.55E-02				+			Natural product
**FLUOROURACIL**	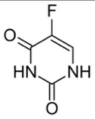	-2.11	1.75E-02	+	+	+				Antimetabolite
**TRYPTOPHAN**	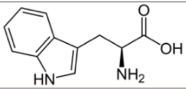	-1.92	2.76E-02	+		+				Other
**ALVOCIDIB**	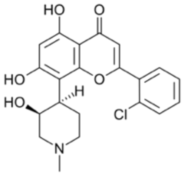	-1.82	3.44E-02	+	+	+				CDK inhibitor

* Table ordered by p-value
