# Supplementary material for: Computational pharmacogenomic screen identifies drugs that potentiate the anti-breast cancer activity of statins
Source: Nat Commun. 2022 Oct 24;13:6323. doi: 10.1038/s41467-022-33144-9 (PMC9592602; doi:10.1038/s41467-022-33144-9)
Supplement: Supplementary file 2 — Description of additional supplementary files [file 41467_2022_33144_MOESM2_ESM.pdf]

Description of Additional Supplementary Files

Supplementary Table 1 - Ranked MVA-DNF compounds. Drug structure, z-score and p-values are indicated. Compounds are ordered by p-value and restricted to Z-score <-1.8.
