# Supplementary material for: Computational pharmacogenomic screen identifies drugs that potentiate the anti-breast cancer activity of statins
Source: Nat Commun. 2022 Oct 24;13:6323. doi: 10.1038/s41467-022-33144-9 (PMC9592602; doi:10.1038/s41467-022-33144-9)
Supplement: Supplementary file 3 — Supplemental table 1 [file 41467_2022_33144_MOESM3_ESM.docx]

| **Hits** | **Drug structure** | **Permutation Test** | | **Currently used in humans** | **Currently used in cancer treatment** | **In clincal trials for cancer treatment** | **Pre-clinical** | **Research tool** | **Exclude** | **Classification** |  |
| --- | --- | --- | --- | --- | --- | --- | --- | --- | --- | --- | --- |
|  |  | **Z-Score** | **P-Value*** |  |  |  |  |  |  |  |  |
| **SELUMETINIB** | 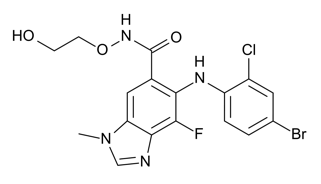 | -3.57 | 1.77E-04 | + |  | + |  |  |  | RAF/MEK inhibitor |  |
| **NELFINAVIR** | 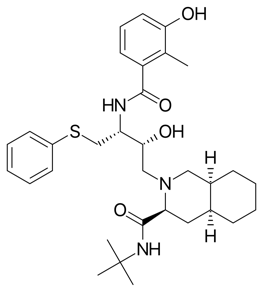 | -3.28 | 5.18E-04 | + |  | + |  |  |  | Antiretroviral |  |
| **MITOXANTRONE** | 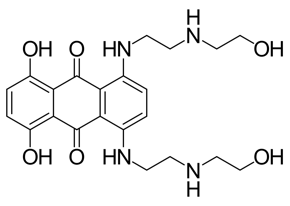 | -3.20 | 6.82E-04 | + | + | + |  |  |  | Anthracycline |  |
| **DOXORUBICIN** | 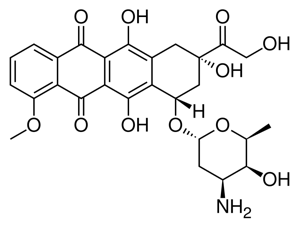 | -3.03 | 1.22E-03 | + | + | + |  |  |  | Anthracycline |  |
| **HONOKIOL** | 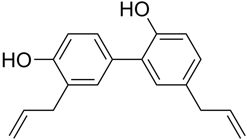 | -2.96 | 1.53E-03 |  |  |  | + |  |  | Natural product |  |
| **CLOTRIMAZOLE** | 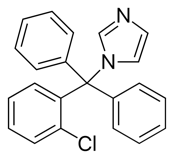 | -2.95 | 1.57E-03 | + |  |  |  |  |  | Antifungal |  |
| **SULFATHIAZOLE** | 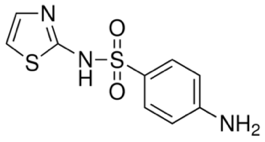 | -2.89 | 1.90E-03 |  |  |  | + |  |  | Antibiotic |  |
| **VEMURAFENIB** | 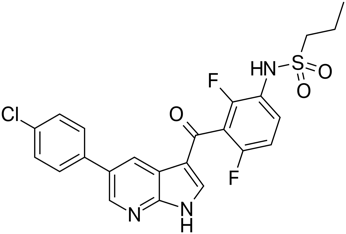 | -2.67 | 3.79E-03 | + | + | + |  |  |  | RAF/MEK inhibitor |  |
| **CHROMOMYCINA3** | 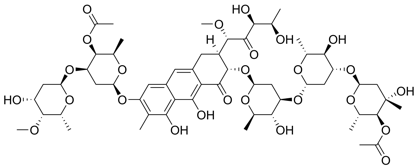 | -2.64 | 4.13E-03 |  |  |  | + | + | Toxin | Antibiotic |  |
| **BACCATINIII** | 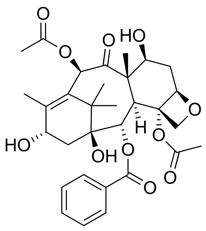 | -2.53 | 5.69E-03 |  |  |  | + |  |  | Natural product |  |
| **NOSCAPINE** | 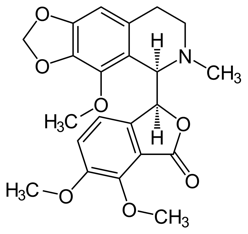 | -2.51 | 6.11E-03 |  |  | + | + |  |  | Natural product |  |
| **METHOTREXATE** | 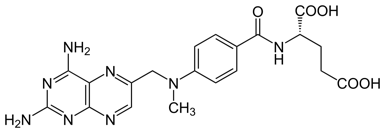 | -2.29 | 1.11E-02 | + | + | + |  |  |  | Antimetabolite |  |
| **CADMIUMCHLORIDE** |  | -2.22 | 1.33E-02 |  |  |  | + | + | Carcinogen | Other |  |
| **RHAMNETIN** | 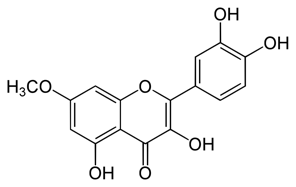 | -2.18 | 1.45E-02 |  |  |  | + |  |  | Natural product |  |
| **PENTAMIDINE** | 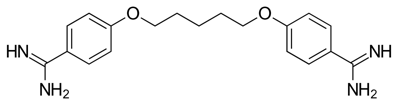 | -2.18 | 1.46E-02 | + |  | + |  |  |  | Antifungal |  |
| **EMODIN** | 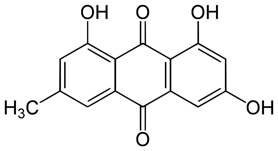 | -2.16 | 1.55E-02 |  |  |  | + |  |  | Natural product |  |
| **FLUOROURACIL** | 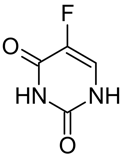 | -2.11 | 1.75E-02 | + | + | + |  |  |  | Antimetabolite |  |
| **TRYPTOPHAN** | 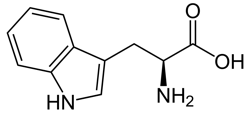 | -1.92 | 2.76E-02 | + |  | + |  |  |  | Other |  |
| **ALVOCIDIB** | 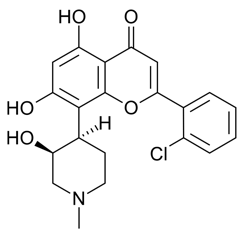 | -1.82 | 3.44E-02 | + | + | + |  |  |  | CDK inhibitor |  |
| **ABIRATERONE** | 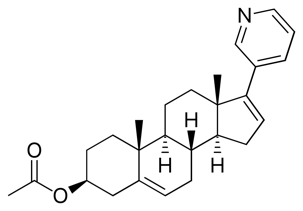 | -1.77 | 3.81E-02 | + | + | + |  |  |  | Antiandrogen |  |
| **THAPSIGARGIN** | 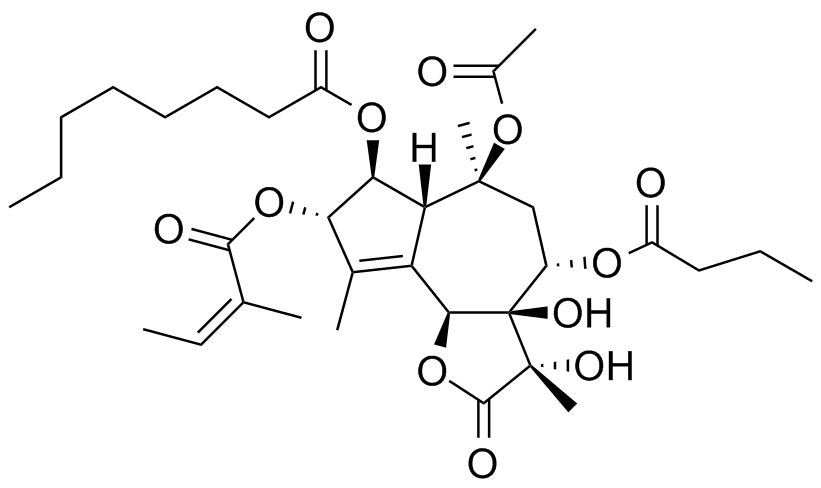 | -1.71 | 4.36E-02 |  |  |  | + |  |  | Other |  |
| **KINETINRIBOSIDE** | 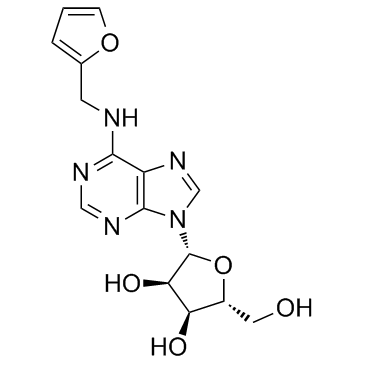 | -1.66 | 4.84E-02 |  |  |  | + |  |  | Other |  |
| **ELLIPTICINE** | 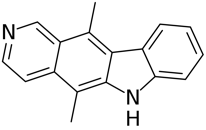 | -1.65 | 4.92E-02 |  |  |  | + |  |  | Natural product |  |
| * Table ordered by p-value | | | | | | | | | | | |
