# Supplementary material for: Computational pharmacogenomic screen identifies drugs that potentiate the anti-breast cancer activity of statins
Source: Nat Commun. 2022 Oct 24;13:6323. doi: 10.1038/s41467-022-33144-9 (PMC9592602; doi:10.1038/s41467-022-33144-9)
Supplement: Supplementary file 4 — Reporting Summary [file 41467_2022_33144_MOESM4_ESM.pdf]

Corresponding author(s): Dr. Linda Z. Penn
Dr. Benjamin Haibe-Kains
Dr. Deena Gendoo

Last updated by author(s): June 22, 2022

Reporting Summary

Nature Portfolio wishes to improve the reproducibility of the work that we publish. This form provides structure for consistency and transparency in reporting. For further information on Nature Portfolio policies, see our Editorial Policies and the Editorial Policy Checklist.

Statistics

For all statistical analyses, confirm that the following items are present in the figure legend, table legend, main text, or Methods section.

n/a	Confirmed	
☐	☒	The exact sample size (*n*) for each experimental group/condition, given as a discrete number and unit of measurement
☐	☒	A statement on whether measurements were taken from distinct samples or whether the same sample was measured repeatedly
☐	☒	The statistical test(s) used AND whether they are one- or two-sided<br>*Only common tests should be described solely by name; describe more complex techniques in the Methods section.*
☐	☒	A description of all covariates tested
☐	☒	A description of any assumptions or corrections, such as tests of normality and adjustment for multiple comparisons
☐	☒	A full description of the statistical parameters including central tendency (e.g. means) or other basic estimates (e.g. regression coefficient) AND variation (e.g. standard deviation) or associated estimates of uncertainty (e.g. confidence intervals)
☐	☒	For null hypothesis testing, the test statistic (e.g. *F*, *t*, *r*) with confidence intervals, effect sizes, degrees of freedom and *P* value noted<br>*Give P values as exact values whenever suitable.*
☒	☐	For Bayesian analysis, information on the choice of priors and Markov chain Monte Carlo settings
☒	☐	For hierarchical and complex designs, identification of the appropriate level for tests and full reporting of outcomes
☐	☒	Estimates of effect sizes (e.g. Cohen's *d*, Pearson's *r*), indicating how they were calculated

Our web collection on statistics for biologists contains articles on many of the points above.

Software and code

Policy information about availability of computer code

Data collection	Computational pharmacogenomic screen data collection and data visualization used the following packages: PharmacoGx package (v1.6.1), iGraph R package, Upset R package, genefu R package, pryr package, SynergyFinder R package, Piano R package.<br>Flow cytometry data were collected on BD LSR II and BD FACS DIVA software v8.<br>Incucyte assay software module was used for cell proliferation assays.<br>Western blot acquisition was done using Odyssey Infrared Imaging System (LI-COR Biotechnology).
Data analysis	Computational pharmacogenomic screen data collection and data visualization used the following packages: PharmacoGx package (version 1.6.1), iGraph R package, UpsetR package, genefu R package, pryr package, SynergyFinder R package, Piano R package. Prism (v8.2.0, GraphPad Software) was used for graph output and statistic analysis.<br>The code and associated tutorial describing how to run the analysis pipeline are publicly available on Github (https://github.com/DGendoo/MVA_DNF). All software dependencies are available on Bioconductor (BioC) or the Comprehensive Repository R Archive Network (CRAN), and have been listed throughout the methods as applicable.<br>There is no unreported algorithm used in this manuscript.<br>Flow cytometry data was analyzed using FlowJo v10.<br>Western blots were analyzed using ImageJ v1.47.

For manuscripts utilizing custom algorithms or software that are central to the research but not yet described in published literature, software must be made available to editors and reviewers. We strongly encourage code deposition in a community repository (e.g. GitHub). See the Nature Portfolio guidelines for submitting code & software for further information.

Data

Policy information about availability of data

All manuscripts must include a data availability statement. This statement should provide the following information, where applicable:

- Accession codes, unique identifiers, or web links for publicly available datasets
- A description of any restrictions on data availability
- For clinical datasets or third party data, please ensure that the statement adheres to our policy

Reference subcellular image library was uploaded to the Image Data Resource (idr0072; https://idr.openmicroscopy.org).

Field-specific reporting

Please select the one below that is the best fit for your research. If you are not sure, read the appropriate sections before making your selection.

☒ Life sciences ☐ Behavioural & social sciences ☐ Ecological, evolutionary & environmental sciences

For a reference copy of the document with all sections, see nature.com/documents/nr-reporting-summary-flat.pdf

Life sciences study design

All studies must disclose on these points even when the disclosure is negative.

Sample size	All experiments involving the analysis of cancer cells were performed with at least three biologically independent samples. Due to the small variability of experimental conditions, these sample sizes are considered sufficient. No statistical tests were used to calculate sample size. Sample sizes are listed in the figure legends and main text.
Data exclusions	No data was excluded.
Replication	Experiments were repeated as indicated above as detailed in the manuscript. All attempts at replication were successful.
Randomization	Randomization is not relevant for in vitro cell line experiments.
Blinding	For cell-based experiments, western blotting and high throughput screening assays blinding was not possible because the experiments were performed by a single researcher.

Reporting for specific materials, systems and methods

We require information from authors about some types of materials, experimental systems and methods used in many studies. Here, indicate whether each material, system or method listed is relevant to your study. If you are not sure if a list item applies to your research, read the appropriate section before selecting a response.

Materials & experimental systems

n/a	Involved in the study	
☐	☒	Antibodies
☐	☒	Eukaryotic cell lines
☒	☐	Palaeontology and archaeology
☒	☐	Animals and other organisms
☐	☒	Human research participants
☒	☐	Clinical data
☒	☐	Dual use research of concern

Methods

n/a	Involved in the study	
☒	☐	ChIP-seq
☐	☒	Flow cytometry
☒	☐	MRI-based neuroimaging

Antibodies

Antibodies used	SREBP-2 (1:250, BD Biosciences, 557037), p44/42 MAPK (ERK1/2) (1:1000, Cell Signaling Technology, 4695), PARP (1:1000, Cell Signaling Technology, 9542L), α-Tubulin (1:3000, Calbiochem, CP06) and E-cadherin (1:1000, Cell Signaling Technology, 3195). IRDye-conjugated secondary antibodies (1:20,000, LI-COR Biosciences, 926-32211 and 926-32210).
Validation	All antibodies used in our study have been validated and detailed information can be found on the website from the commercial providers. Specific signal for SREBP-2 has been previously knockdown validated (PMID: 31023626).

Eukaryotic cell lines

Policy information about cell lines

Cell line source(s)	BC cell line panel was a generous gift from Dr. Benjamin Neel (Department of Medicine at NYU Grossman School of Medicine). Normal murine mammary gland (NMuMG) cells were a a gift of J. Wrana, Lunenfeld-Tanenbaum Research Institute, Toronto, Canada and cultured in DMEM (Gibco), containing 10 µg/ml bovine insulin (Sigma). HEK293T were a gift from Frank Graham at McMaster University, Hamilton, Canada and grown in DMEM.
Authentication	Cell lines were not authenticated.
Mycoplasma contamination	All cell lines tested negative for mycoplasma contamination.
Commonly misidentified lines (See ICLAC register)	No commonly misidentified cell lines were used in this study.

Human research participants

Policy information about studies involving human research participants

Population characteristics	Whole blood was collected into 3.2% sodium citrate (Becton Dickinson) at a ratio of 9:1 (vol:vol) from healthy donors who provided written consent and were compensated financially (REB: Hamilton Integrated Research Ethics Board #4804).
Recruitment	*Describe how participants were recruited. Outline any potential self-selection bias or other biases that may be present and how these are likely to impact results.*
Ethics oversight	REB: Hamilton Integrated Research Ethics Board #4804

Note that full information on the approval of the study protocol must also be provided in the manuscript.

Flow Cytometry

Plots

Confirm that:

- ☒ The axis labels state the marker and fluorochrome used (e.g. CD4-FITC).
- ☒ The axis scales are clearly visible. Include numbers along axes only for bottom left plot of group (a 'group' is an analysis of identical markers).
- ☒ All plots are contour plots with outliers or pseudocolor plots.
- ☒ A numerical value for number of cells or percentage (with statistics) is provided.

Methodology

Sample preparation	Cells were seeded at 2.5x10^5 cells/plates and treated the next day as indicated. After 72 hours, cells were fixed in 70% ethanol for >24 h, stained with propidium iodide and analyzed by flow cytometry for the sub-diploid (% pre-G1) DNA population as a measure of cell death as previously described in Clendening, J. W. et al. Exploiting the mevalonate pathway to distinguish statin-sensitive multiple myeloma. Blood 115, 4787–4797 (2010).
Instrument	Flow cytometry data were collected on BD LSR II using BD FACS DIVA software.
Software	FlowJo_V10 software was used to analyze data.
Cell population abundance	At least 10,000 events were collected for each sample.
Gating strategy	Initial cell population gating (FSC-Area vs FSC-Height) was adopted to make sure doublet exclusion and only single cell were used for analysis.

☒ Tick this box to confirm that a figure exemplifying the gating strategy is provided in the Supplementary Information.
